# Determination of optimal sampling time of grape embryo rescue based on near infrared spectroscopy combined with machine learning

**DOI:** 10.1016/j.plaphe.2025.100044

**Published:** 2025-05-29

**Authors:** Fuqiang Wang, Lu Bian, Zhanzhan Zhan, Yao Chen, Chendong Ling, Han Guo, Yueqi Gai, Guotian Liu, Tengfei Xu, Yuejin Wang, Yan Xu, Yingqiu Huo

**Affiliations:** a State Key Laboratory for Crop Stress Resistance and High-Efficiency Production, Northwest A&F University, Yangling, 712100, China; b Key Laboratory of Horticultural Plant Biology and Germplasm Innovation in Northwest China, Ministry of Agriculture and Rural Affairs, Yangling, 712100, China; c College of Horticulture, Northwest A&F University, Yangling, 712100, China; d Key Laboratory for Agricultural Internet of Things, Ministry of Agriculture and Rural Affairs, Yangling, 712100, China; e College of Information Engineering, Northwest A&F University, Yangling, 712100, China

**Keywords:** Seedless grape, Sampling time, Pericarp puncture hardness, Near infrared spectroscopy, Machine learning

## Abstract

Grape embryo rescue technology is currently the primary method for breeding new seedless grape cultivars. The timing of berry sampling directly impacts the efficacy of this technique. Therefore, achieving efficient, accurate, and non-destructive determination of the optimal sampling time for seedless grape embryo rescue breeding has long been a challenge. This study collected near-infrared spectral data and data on 19 physiological indicators from 2940 grape berries of six grape cultivars at six sampling times to construct a baseline dataset. Remarkably, it was discovered for the first time that pericarp puncture hardness (PPH) is closely associated with the embryo development rate of seedless grape. Subsequently, the optimal sampling times for 'Flame Seedless', 'Ruby Seedless', and 'Jingzaojing' were determined when their PPH reached 720 ​± ​20 ​g, 990 ​± ​20 ​g and 633 ​± ​20 ​g, respectively. Then, a total of 840 models for PPH recognition were established and assessed based on their coefficient of determination (*R*^2^) and root mean square error (RMSE). The optimal recognition models for three seedless grape cultivars suitable for embryo rescue—'Flame Seedless', 'Ruby Seedless', and 'Jingzaojing'—were identified as follows: D1+PLSR (*R*^2^ ​= ​0.94, RMSE ​= ​42.26), D1+MLR (*R*^2^ ​= ​0.79, RMSE ​= ​66.31) and D1+PLSR (*R*^2^ ​= ​0.93, RMSE ​= ​47.9). Utilizing the established D1+PLSR or D1+MLR models for PPH, a non-destructive and precise method for sampling seedless grapes during embryo rescue was introduced for the first time. This approach led to a notable increase in the embryo development rate by 15 ​% and enhanced the plantlet rate by 14 ​%. Overall, our proposed strategy provides new perspectives for accelerating the breeding process of new seedless grape cultivars.

**Table:** Abbreviations

DAP: Days after pollination	D1: First derivative
DAF: Days after flowering	D2: Second derivative
NIRS: Near-infrared spectroscopy	EDF: Exponentially decreasing function
AI: Artificial intelligence	RMSECV: The root mean square error of cross-validation
SSC: Soluble solid content	PLSR: Partial least squares regression
L∗: Lightness value	SVR: Support vector regression
a∗: Red-green value	RR: Ridge regression
b∗: Yellow-blue value	LASSO: Least absolute shrinkage and selection operator
CIRG: Color index for red grapes	MLR: Multiple linear regression
H: Berry hue angle	RFR: Random forest regression
C: Berry color chroma	XGBoost: Extreme gradient boosting
6-BA: 6-Benzylaminopurine	LARS: Least angle regression
IAA: Indole-3-acetic acid	*R*^2^: The coefficient of determination
IBA: Indole-3-butytric acid	RMSE: Root mean square error
WPM: Wood plant medium	S1: E-L-32
CA: Correlation analysis	S2: E-L-33
PCA: Principal component analysis	S3: E-L-34
PC1: First principal component	S4: E-L-35
PC2: Second principal component	S5: E-L-36
PC3: Third principal component	S6: E-L-37
GRA: Grey relation analysis	BLD: Berry longitudinal diameter
FS: Flame Seedless	BTD: Berry transverse diameter
RS: Ruby Seedless	BSI: Berry shape index
JZJ: Jingzaojing	BFW: Berry fresh weight
MH: Muscat Hamburg	OLD: Ovule longitudinal diameter
PN: Pinot Noir	OTD: Ovule transverse diameter
SM: Shine Muscat	OSI: Ovule shape index
SG: Savitzky-Golay filter	OFW: Ovule fresh weight
MMN: Min-Max normalization	PPH: Pericarp puncture hardness
SNV: Standard normal variate transformation	PBD: Pericarp break distance
MSC: Multiplicative scatter correction	PS: Pericarp stiffness
CARS: Competitive adaptive reweighted sampling	SF: Sarcocarp firmness

## 1 Introduction

Seedless grapes are popular not only for fresh consumption but also for drying and juice production, making them highly favored by both consumers and producers [1,2]. Consequently, seedlessness in grapes holds significant economic value and is an important target trait in breeding programs [[3], [4], [5], [6], [7]]. Based on the pollination mechanism and seed presence, seedless grapes can be classified into parthenocarpic and pseudo-parthenocarpic types [8]. Parthenocarpic seedless grapes are those that develop into seedless berries without fertilization. In contrast, pseudo-parthenocarpic seedless grapes undergo normal fertilization to form zygotic embryos; however, during seed development, the embryo growth ceases, resulting in berries with only trace amounts of seeds. This type is also known as stenospermocarpic grapes [9]. Currently, more than 85 ​% of the seedless grape cultivars used in breeding research and production are of the pseudo-parthenocarpic type. These seedless grape cultivars are also used as the maternal parent in hybridization for grape embryo rescue breeding. The grape embryo rescue technique is based on the principle of cellular totipotency. It involves extracting ovules before embryo abortion occurs, followed by culturing them in a medium that promotes embryo development and seedling growth [10]. Since its successful implementation in 1982, this technique has gained popularity in seedless grape breeding due to its significant advantages. These advantages include effectively addressing the issues related to sexual reproduction when using seedless grapes as the maternal parent in hybridization, increasing the proportion of seedless hybrids, and shortening the breeding cycle [11,12]. Researchers worldwide have developed 150 new seedless grape cultivars using embryo rescue techniques (https://www.uspto.gov/and https://www.cnki.net/). However, the efficiency of embryo rescue depends on many factors, including parental genotype, sampling time, culture medium type and additives, and cultivation conditions [13]. Notably, sampling time is significantly negatively correlated with embryo development rate [14]. In other words, collecting ovules at the optimal sampling time for embryo culture leads to a higher embryo development rate. Therefore, accurately and non-destructively determining the optimal sampling time for grape embryo rescue is of great research significance and practical application value for improving the efficiency of seedless grape breeding.

To determine the optimal sampling time in grape embryo rescue breeding, existing research primarily relies on DAP or DAF [[15], [16], [17], [18]]. The optimal sampling time varies significantly among different seedless grape cultivars. For early-maturing cultivars, the sampling time is approximately DAP 30, while for mid-to late-maturing cultivars, it ranges from DAP 40 to 50. Additionally, the optimal sampling time can vary considerably even within the same variety due to factors such as growth year, climatic conditions, and cultivation management [19]. For example, researchers have identified the optimal sampling time for 'Flame Seedless' in 2005, 2013, and 2021 as DAP 37, DAP 39, and DAP 42, respectively [[20], [21], [22]]. It is evident that using days after pollination as a metric is inherently unstable for determining the optimal sampling time, preventing subsequent research from directly taking previous findings as the optimal sampling time for seedless grape cultivars in the actual year. Grey et al. [23] suggested that 1–2 days before berry color change is the optimal sampling time for embryo rescue, as the berry is in a state of incomplete softening at that stage, making it easier to separate the ovules from the flesh. Meng et al. [24] proposed that during the berry softening period, embryo development is relatively advanced, enabling the acquisition of hybrid plantlets without excessive auxin application. Therefore, this period can be regarded as the optimal sampling time. Moreover, Wang et al. [25] conducted cytological observations of hybrid embryos from both seedless and seeded grapes and found that successful embryo rescue was more likely when the ovule weight and length-to-width ratio reached their maximum values. These factors can thus serve as valuable reference indicators for determining the optimal sampling time. Therefore, to determine the optimal sampling time for embryo rescue, researchers have used not only days after pollination but also the developmental state of the berries or ovules as reference indicators. Since these parameters are consistent for each grape variety [26], assessing the optimal sampling time based on the actual developmental and physicochemical indicators of the berry can effectively mitigate the instability caused by differences in variety, climate, year, and cultivation conditions. However, using the developmental state of the berries or ovules as a reference indicator for the optimal sampling time presents several issues: not only is the berry structure compromised, leading to the waste of experimental materials, but there is also no precise evaluation metric to define the developmental state. Therefore, developing accurate and non-destructive methods to determine the optimal sampling time for embryo rescue is essential for improving the efficiency of embryo rescue breeding and is a necessary requirement for adaptation to intelligent grape breeding. In recent years, NIRS combined with AI technology has demonstrated significant potential for the rapid, non-destructive and accurate detection of agricultural product quality and safety [[27], [28], [29], [30], [31], [32], [33]]. For instance, researchers have used NIRS in conjunction with PLSR algorithms to non-destructively predict SSC, firmness, pH, and seedlessness in mature grapes [34,35]. This technique has also been applied to determine the maturity of pomegranates [36], the astringency levels of persimmons [37], and the dry matter and soluble solid content in kiwifruits [38]. Currently, NIRS technology has been primarily applied to quality assessment during the maturity phase of grapes, but no research has reported its use for assessing attributes in seedless grapes during the hardening to softening stages.

In line with the above requirements, this study aimed to investigate the changes in physiological indicators of berries and ovules during grape development, combined with embryo rescue breeding technology, to identify indicators strongly correlated with the optimal sampling time for seedless grape embryo rescue. Additionally, by scanning grapes using a near-infrared spectrometer, this study aimed to establish the best model correlating near-infrared spectra with berry physiological indicators. The findings provide a non-destructive approach for precise embryo rescue sampling and improve the efficiency of seedless grape embryo rescue breeding.

## 2 Materials and methods

### 2.1 Plant materials and sampling

The experimental materials were obtained from the grape germplasm repository at Northwest A&F University in Yangling, Shaanxi Province (108°4′27.95″ E, 34°16′56.24″ N). Several seven-year-old cultivars were selected, including 'Flame Seedless' (*V. vinifera*, seedless), 'Ruby Seedless' (*V. vinifera*, seedless), 'Jingzaojing' (*V. vinifera*, seedless), 'Pinot Noir' (*V. vinifera*, seeded), 'Muscat Hamburg' (*V. vinifera*, seeded), and 'Shine Muscat' (*V. vinifera* ​× ​*V. labrusca*, seeded). Grapevines were cultivated under rain-shelter canopies using a 'V'-shaped horizontal trellis system, along with systematic water and fertilizer management practices. Sampling was conducted at six time points, ranging from E-L-32 to E-L-37 stage, following the sampling standards established by the E-L system for grape growth [26]. Sampling was carried out daily between 8:00 and 9:00 a.m. To preserve biological integrity, harvested grape samples were immediately transferred to pre-chilled insulated containers (4 ​°C). All necessary physiological indicator measurements and spectral data acquisitions were completed within a strict 4-h window after collection.

### 2.2 Experimental methods

#### 2.2.1 Determination of size and weight of grape berries and ovules

The longitudinal and transverse diameters of grape berries and ovules were measured using a caliper, and the fresh weights of the berries and ovules were determined using a precision electronic balance (0.001 ​g). Healthy berries of uniform size and without visible defects were systematically selected. To ensure measurement accuracy, the cumulative weight of each variety at each stage (using at least 10 samples) was determined, and the mean weight was calculated to determine the weight of individual berries or ovules. This procedure was repeated three times to ensure biological replication.

#### 2.2.2 Determination of NIRS data of grape berry

The NIRS data of grape berries were collected using a near-infrared spectrometer (AgriSpec® 2 Hi-Res, ASD, USA). The spectral range covered 350–1830 ​nm, with the probe positioned in close contact with the equatorial region of the grape berry. Before each data collection session, white balance correction was performed to ensure accuracy. Each grape berry was measured three times, and the average of these readings was taken to obtain a single sample data point. All spectral data were collected in a controlled indoor environment with a stable light source (25.0 ​± ​1 ​°C, 50.0 ​± ​5 ​% RH)). For each variety at each stage, a minimum of 200 berries, exhibiting uniform size and free from lesions or damage, were randomly selected.

#### 2.2.3 Determination of grape berry color

After spectral scanning, the berries were sequentially numbered. A color difference meter (CR-400, Konica Minolta, Japan) was then used to measure three points on the equatorial region of each berry. This allowed for the evaluation of L∗, a∗, and b∗. The CIRG for each berry was calculated using the formula (CIRG=(180-H)/(L∗+C)), where the berry hue angle (H ​= ​arctan(b∗/a∗)), and the berry color chroma (C ​= ​[(a∗)^∧^2+(b∗)^∧^2]^∧^0.5) [39].

#### 2.2.4 Determination of grape berry texture

After being scanned by the color difference meter, the berries were individually subjected to a puncture test following their numbering sequence. This test evaluated the pericarp puncture hardness, pericarp stiffness, and sarcocarp firmness [40,41]. To minimize testing errors, all measurements were conducted under controlled conditions (25.0 ​± ​1 ​°C, 50.0 ​± ​5 ​% RH) in a temperature-controlled environment. The same operator performed all measurements using the same instrument. The testing instrument used was the TA.XT plus Texture Analyzer (Stable Micro Systems, Surrey, UK). The testing parameters included a P/2 needle probe with a diameter of 2 ​mm, a pre-test speed of 2 ​mm/s, a penetration speed of 2 ​mm/s, a post-test speed of 10 ​mm/s, a penetration depth of 5 ​mm, and a trigger load of 5 ​g. During parameter configuration, care was taken to ensure the required puncture depth was achieved while avoiding contact between the probe and grape seeds, thereby preventing any impact on the test results.

#### 2.2.5 Determination of SSC in grape berry

The SSC of grape berries was measured using a glucose-acid meter (PAL-BX-ACID2, ATAGO, Japan), with a measurement range of 0.0–60.0 % and an accuracy of ± 0.2 ​%. For each variety at each stage, a minimum of 10 berries were tested collectively, and the average value was recorded as a biological replicate. This process was repeated three times.

#### 2.2.6 Procedure of grape embryo rescue technique

(1) Cross-pollination

Using 'Flame Seedless', 'Ruby Seedless', and 'Jingzaojing' as female parents, natural cross-pollination was performed to obtain berries for the correlation analysis of physiological indices. 'Flame Seedless' and 'Ruby Seedless' served as female parents, while 'Muscat Hamburg' and 'Shine Muscat' served as male parents, resulting in four hybrid combinations: 'Flame Seedless' ​× ​'Muscat Hamburg', 'Flame Seedless' ​× ​'Shine Muscat', 'Ruby Seedless' ​× ​'Muscat Hamburg', and 'Ruby Seedless' ​× ​'Shine Muscat'. The resulting hybrid berries were used for model verification and application.

(2) Configuration of medium

Embryo development culture medium: MM3 ​+ ​Sucrose 60 ​g/L ​+ ​Activated Charcoal 3 ​g/L ​+ ​Agar 7 ​g/L ​+ ​IAA 1 ​mg/L ​+ ​Hydrolyzed Casein 0.5 ​g/L ​+ ​Inositol 0.1 ​g/L, pH ​= ​5.8–6.0.

Embryo germination medium: WPM ​+ ​Sucrose 20 ​g/L ​+ ​Activated Charcoal 1.5 ​g/L ​+ ​Agar 7 ​g/L + 6-BA 0.2 ​mg/L ​+ ​Inositol 0.1 ​g/L, pH ​= ​5.8–6.0.

Subculture medium: WPM ​+ ​Sucrose 30 ​g/L ​+ ​Activated Charcoal 1.5 ​g/L ​+ ​Agar 7 ​g/L ​+ ​IBA 0.2 ​mg/L, pH ​= ​5.8–6.0.

(3) Specific operation method of embryo rescue [13].

Material Disinfection: Individual berries were cut using scissors, and their quantity was recorded before transferring them to a beaker. The beaker was covered with a net bag, and water was circulated to gently swirl the berries for 3–4 ​h. Subsequently, under a sterile laminar flow hood, the berries were transferred to sterilized glass vials and immersed in 75 ​% ethanol for 30 ​s. The ethanol was then discarded, and the berries were rinsed twice with sterile water. A 1.5 ​% sodium hypochlorite solution was added to cover the berries, which were then allowed to soak for 30 ​min. After discarding the sodium hypochlorite solution, the berries were rinsed three times with sterile water.

Inoculation: Under a sterile laminar flow hood, the berries were cut open with a surgical knife to extract the ovules. Ovules larger than 2 ​mm were placed onto the embryo development medium and incubated in a tissue culture room under dark conditions at 25 ​°C for 8–10 weeks.

Embryo Separation: Under a sterile laminar flow hood and with the aid of a dissecting microscope, the ovules were carefully incised with a surgical knife, and the immature embryos were extracted using dissecting needles. These embryos were transferred to embryo germination medium, and the embryo development rate was calculated as (%) = (number of developed embryos/number of cultured ovules) ​× ​100. The embryos were then uniformly placed in the tissue culture room under a light regime of 16 ​h light and 8 ​h darkness at 25 ​°C.

Subculture Proliferation: After 8 weeks of culture in the embryo germination medium, the plantlet rate was calculated as (%) = (number of plantlets/number of cultured ovules) ​× ​100. To ensure a high survival rate of hybrid plantlets, robust stem segments were excised and transferred to the subculture medium for propagation. Optimal timing was selected for the acclimatization and transplantation of plantlets. The culture conditions were maintained at 16 ​h of light and 8 ​h of darkness at 25 ​°C.

#### 2.2.7 Data analysis

Basic statistical analyses and calculations of univariate quadratic regression equations were performed using Excel 2021. One-way ANOVA and Duncan's test were used for the comparative analysis of each treatment, with a significance level set at *P* ​≤ ​0.05. SPSS 26.0 was used for CA, PCA, and GRA of the measurement results for various indicators [42,43], while Origin 2018 was used for graphing.

### 2.3 Construction of non-destructive grape berry AI models

#### 2.3.1 Classification of NIRS dataset of grape berries

The NIRS dataset of grape berries was categorized into four main groups, comprising a total of 15 subgroups based on all data, including different cultivars, batches, and characteristics. For each group, the data were randomly partitioned into a training set and a testing set, with 80 ​% and 20 ​% of the samples assigned, respectively (Table S1).

#### 2.3.2 Analysis of data outliers

To ensure data reliability during model construction and enhance the robustness of model outputs against interference, this study implements a boxplot-based methodology for robust outlier detection in raw datasets. Leveraging the nonparametric statistical characteristics of the interquartile range (IQR), observations falling outside the [Q1 - 1.5 ​× ​IQR, Q3 ​+ ​1.5 ​× ​IQR] range are systematically identified as statistically significant anomalies.

#### 2.3.3 Pretreatment of NIRS data

During the acquisition of NIRS data, environmental or instrumental variations often introduce noise into the raw spectral signals. To enhance the signal-to-noise ratio and extract meaningful information from the spectral data, this study employed six preprocessing methods: SG, MMN, SNV, MSC, D1, and D2.

#### 2.3.4 Feature band extraction based on CARS

NIRS data frequently exhibit significant redundant information, which elevates computational complexity and adversely affects model performance [44]. This study used the CARS algorithm to extract feature bands from the full spectral dataset. The CARS algorithm emulates the ‘Survival of the Fittest’ principle from Darwinian evolutionary theory by integrating Monte Carlo sampling with the PLSR model [45]. During feature band extraction, a ten-fold cross-validation method was applied [46], with 50 sampling iterations performed on the NIRS data of grape berry samples. In each iteration, 80 ​% of the samples were assigned to the training set, and the remaining 20 ​% were constituted the testing set for PLSR model construction. The absolute values of the PLSR coefficients for each spectral band were systematically recorded during all sampling procedures. The retention rates of spectral bands from each iteration were calculated via the exponential decreasing function (EDF), thereby enabling the elimination of bands exhibiting lower contribution weights.

Based on the predefined number of iterations, the PLSR model was iteratively optimized, and the RMSECV was calculated, as formulated in Equation (1), where $y_{j}$ denotes the measured value, $y_{j}^{\prime }$ represents the predicted value during *K*-fold cross-validation, and *h* is the total number of cross-validation folds (set to 10 in this study). The spectral band subset producing the minimal RMSECV value was identified as the optimal feature band subset for subsequent analysis.

(1) $$\text{RMSECV}=\sqrt{\frac{\sum_{j=1}^{h}{\left(y_{j}-y_{j}^{\prime }\right)}^{2}}{h}}$$

#### 2.3.5 Model construction

Based on the feature bands extracted through the CARS method, eight regression algorithms were employed to establish AI models for grape berry analysis. These algorithms included PLSR, SVR, RR, LASSO, MLR, RFR, XGBoost, and LARS [47,48].

#### 2.3.6 Model evaluation

The performance of the model in predicting grape berry attributes was evaluated using the *R*^2^ and RMSE. The calculation formulas for $R^{2}$ and RMSE are presented in Equations (2), (3), respectively, where $y^{\prime }$ represents the predicted value, y denotes the measured value, $\bar{y}$ is the mean of the measured values, and C is the total number of samples. $R^{2}$ quantifies the proportion of variance in the dependent variable that is explained by the independent variable, reflecting the ability of the model to accurately predict the data. A value of $R^{2}$ closer to 1 indicates stronger predictive performance. RMSE measures the magnitude of deviation between the predicted values and the measured values, with smaller RMSE indicating higher predictive accuracy and lower model error.

(2) $$R^{2}=1-\frac{\sum_{t=1}^{C}\frac{{\left(y-y^{\prime }\right)}^{2}}{C}}{\sum_{t=1}^{C}\frac{{\left(y-\bar{y}\right)}^{2}}{C}}$$

(3) $$\text{RMSE}=\sqrt{\sum_{t=1}^{C}\frac{{\left(y^{\prime }-y\right)}^{2}}{C}}$$

## 3 Results

### 3.1 Physiological changes in the grape berries and ovules of different cultivars

**Fig. 1:**
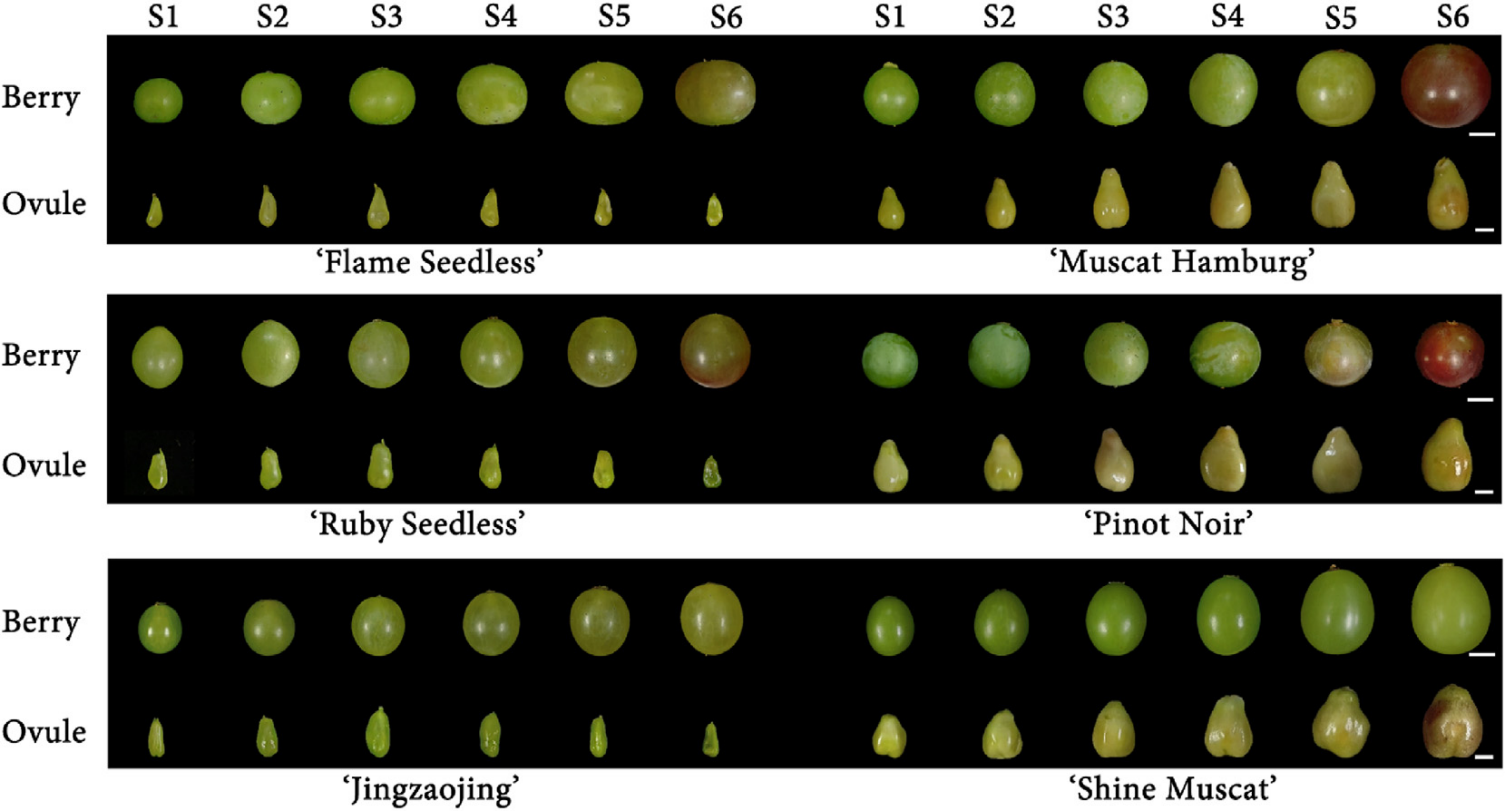
Phenotypic changes in grape berries and ovules across developmental stages. Developmental stages S1-S6 correspond to stages 32–37 of the E-L system for grapevine growth. Scale bars: long bar ​= ​6 ​mm; short bar ​= ​2 ​mm.

**Fig. 2:**
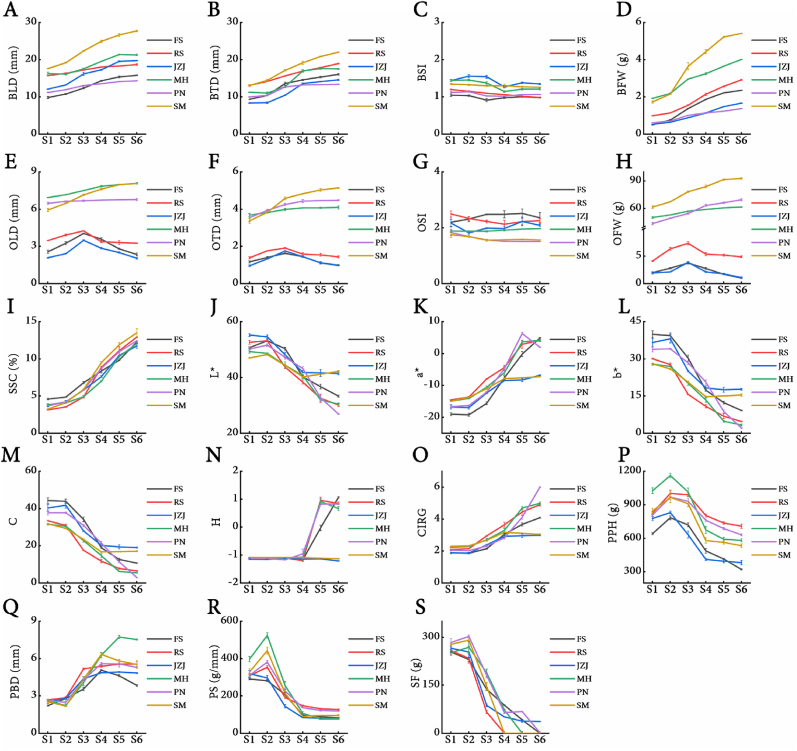
Developmental stage-dependent changes in physiological indices of grape berries.

As the grapes continued to grow, the length and width of berries from six grape cultivars showed an initial slow increase, followed by a rapid rise and eventual stabilization (Fig. 1, Fig. 2A-B). Specifically, the berry dimensions increased slowly from E-L-32 to E-L-33, followed by a rapid increase starting from E-L-34 until stabilization after E-L-36. Except for ‘Shine Muscat’, which showed minimal change in BSI, the remaining five grape cultivars exhibited a trend of gradual decline in their BSI until E-L-35, after which it stabilized (Fig. 2C). Regarding BFW, all six cultivars initially showed a slow increase, followed by a rapid rise starting at E-L-34 (Fig. 2D). Regarding ovule dimensions, the three seeded grape cultivars showed a rapid increase until E-L-34, after which they stabilized (Figs. 1, 2E-F). In contrast, the three seedless grape cultivars exhibited an initial upward trend, peaking at E-L-34 before declining continuously. The OSI for all six grape cultivars remained relatively stable (Fig. 2G). Regarding fresh weight, the three seeded cultivars showed a rapid increase that slowed down later, while the three seedless cultivars exhibited an initial increase followed by a decrease, reaching maximum values at E-L-34. This suggests that the ovules of seedless grapes reach their maximum size during E-L-34, followed by a rapid process of withering and degeneration (Fig. 2H).

The SSC of berries from the six grape cultivars showed an initial gradual increase, followed by a rapid rise starting at the E-L-34 stage (Fig. 2I). This trend suggests that the E-L-34 stage marks the onset of rapid accumulation of soluble organic matter in grape berries. The L∗, b∗, and C values of the berries showed a declining trend (Fig. 2J-M). The a∗ values of these grape cultivars initially increased before stabilizing. Notably, the two green-skinned cultivars, ‘Jingzaojing’ and ‘Shine Muscat’, reached stability earlier than the other four red-skinned cultivars and exhibited lower a∗ values (Fig. 2K). Regarding the H values, ‘Jingzaojing’ and ‘Shine Muscat’ maintained stability, while ‘Flame Seedless’, ‘Ruby Seedless’, ‘Muscat Hamburg’, and ‘Pinot Noir’ were initially stable but showed a sharp increase starting from the E-L-35 stage (Fig. 2N). Regarding the CIRG values, all six cultivars initially remained stable and then increased continuously from the E-L-34 stage. By the E-L-35 stage, ‘Jingzaojing’ and ‘Shine Muscat’ had stabilized in terms of their CIRG values, while the other cultivars continued to increase (Fig. 2O).

The PPH of the six grape cultivars initially increased, reaching a maximum value at the E-L-33 stage, followed by a rapid decline starting at the E-L-34 stage (Fig. 2P). The pericarp break distance for these cultivars initially remained stable, increased sharply from the E-L-33 stage, and stabilized after the E-L-34 stage (Fig. 2Q). The pericarp stiffness of all six cultivars also showed an initial increase, peaking at the E-L-33 stage, followed by a rapid decline starting from the E-L-34 stage and stabilizing by the E-L-36 stage (Fig. 2R). The flesh firmness showed a trend similar to pericarp stiffness; however, by the E-L-35 stage, flesh firmness values below 100 ​g were recorded for all cultivars, indicating that the flesh had softened, which is unfavorable for the embryo rescue of seedless grapes (Fig. 2S).

### 3.2 PCA of different physiological indices

PCA was conducted on 19 physiological indices, including berry dimensions, fresh weight, color parameters, and texture attributes, for three seedless grape cultivars. The analysis identified the three principal component factors with the highest eigenvalues, which collectively explaining 89.36 ​% of the total variance (Table S2). PC1 had an eigenvalue of 11.15 and a variance contribution rate of 58.66 ​%, and it was primarily influenced by indices such as BTD, BFW, SSC, CIRG, and pericarp break distance. PC2 had an eigenvalue of 3.82 and a variance contribution rate of 20.12 ​%, with significant loadings from PPH, OLD, OTD, and OFW. PC3 had an eigenvalue of 2.01 and a variance contribution rate of 10.58 ​%, primarily derived from the BSI and BLD. A comprehensive analysis of the top three principal components suggested that PPH, OFW, OLD, OTD, BSI, and SSC were key physiological determinants of embryo degeneration process in seedless grapes.

### 3.3 Effects of sampling period on the embryo development of seedless cultivars

**Fig. 3:**
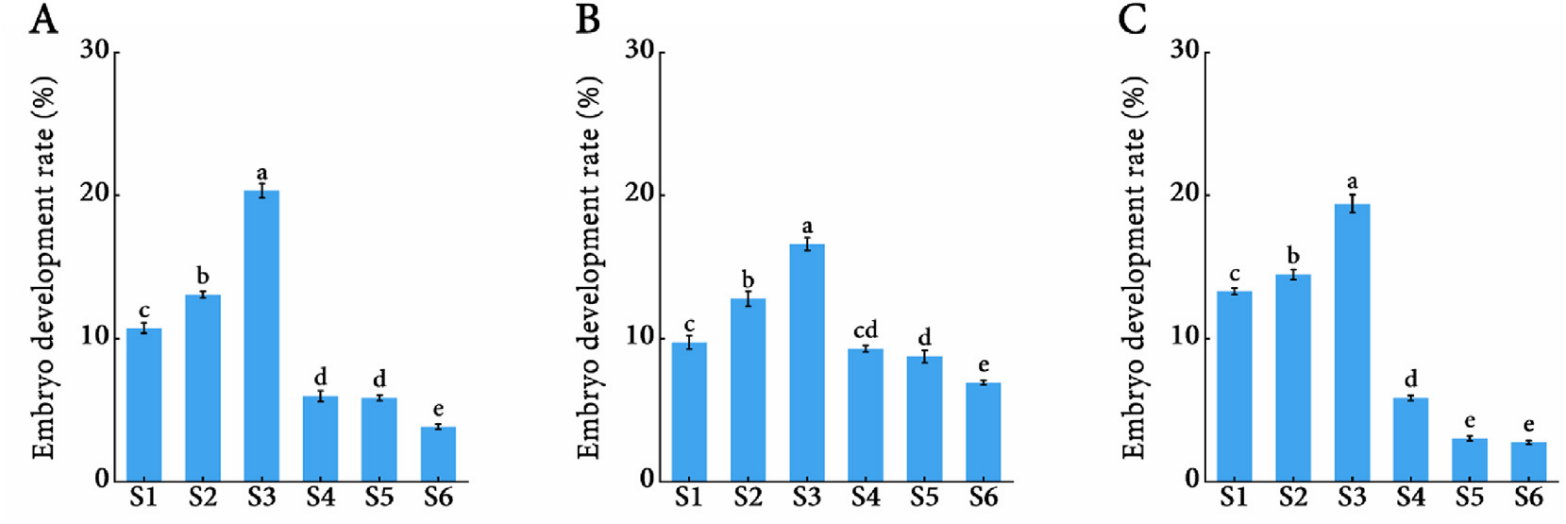
Statistical analysis of the embryo development rates of different seedless grape cultivars after cultivation at different sampling stages. (A) 'Flame Seedless'; (B) 'Ruby Seedless'; (C) 'Jingzaojing'.

In embryo rescue experiments, ovules were collected from naturally pollinated berries of ‘Flame Seedless’, ‘Ruby Seedless’ and ‘Jingzaojing’ cultivars at six developmental stages (E-L-32 to E-L-37). The embryo development rates were quantified for each seedless grape cultivar at different sampling times. For the ‘Flame Seedless’ cultivar, the highest embryo development rate was observed at the E-L-34 stage (20.34 ​%), followed by the E-L-33 stage (13.07 ​%), while the lowest rate was recorded at the E-L-37 stage (3.85 ​%) (Fig. 3). Sampling at the E-L-34 enhanced the embryo development rate by 7.27–16.49 ​% compared to other stages. Similarly, ‘Ruby Seedless’ and ‘Jingzaojing’ showed maximal embryo development at E-L-34, with rate increases of 3.85–7.88 ​% and 4.95–16.69 ​%, respectively, relative to other stages. It was generally concluded that the period with the highest embryo development rates represents the optimum sampling time. Thus, the E-L-34 stage was identified as the optimal sampling period for embryo rescue in 'Flame Seedless', 'Ruby Seedless' and 'Jingzaojing' cultivars, enhancing the embryo development rates by 3.85–16.69 ​%.

### 3.4 Correlation analysis and grey correlation degree analysis between different physiological indices and embryo development rates in seedless grapes

In this study, data from 19 physiological indices and the embryo development rates of three seedless grape cultivars were standardized before performing correlation analysis and grey relational analysis. The correlation analysis revealed that the PPH, BLD and OFW of 'Flame Seedless' cultivar showed significantly positively correlations with its embryo development rate. Among these, PPH exhibited the strongest correlation (0.85, *P* ​≤ ​0.05). For 'Ruby Seedless', significant positive correlations were found between PPH, OLD, OTD and OFW and embryo development rate, with OLD having the highest correlation coefficient (0.99, *P* ​≤ ​0.05), followed by PPH (0.92, *P* ​≤ ​0.05). In 'Jingzaojing', PPH, BSI and OFW were significantly positively correlated with embryo development rate, while BTD, BFW and SSC showed significant negative correlations (Table 1). By averaging correlation coefficients of the 19 physiological indices across the three seedless grape cultivars, only PPH (0.86, *P* ​≤ ​0.05) and OFW (0.84, *P* ​≤ ​0.05) were significantly positively correlated with embryo development rates, with PPH ranking highest. These findings suggested that PPH had the strongest correlation with embryo developmental status. Grey relational analysis further confirmed this pattern, showing PPH (γ ​= ​0.84) and OFW (γ ​= ​0.80) as the top two indicators linked to embryo development (Table S3).

**Table 1:** Correlation coefficient of different physiological indices and embryo development rates of different seedless grape cultivars.

Code	Index	Correlation coefficient			MVCC	Ranking
		Flame Seedless	Ruby Seedless	Jingzaojing		
K1	Berry longitudinal diameter	−0.63	−0.45	−0.73	−0.60	11
K2	Berry transverse diameter	−0.47	−0.49	−0.84^a^	−0.60	12
K3	Berry shape index	−0.39	0.46	0.84^a^	0.30	17
K4	Berry fresh weight	−0.60	−0.61	−0.82^a^	−0.67	9
K5	Pericarp puncture hardness	0.85^a^	0.92^a^	0.81^a^	0.86^a^	1
K6	Pericarp break distance	−0.47	−0.21	−0.59	−0.42	16
K7	Pericarp stiffness	0.66	0.38	0.64	0.56	14
K8	Sarcocarp firmness	0.58	0.30	0.60	0.49	15
K9	Soluble solids content	−0.65	−0.68	−0.84^a^	−0.72	5
K10	CIRG value	−0.77	−0.59	−0.79	−0.72	6
K11	L∗ value	0.82^a^	0.53	0.76	0.70	7
K12	a∗ value	−0.75	−0.56	−0.77	−0.69	8
K13	b∗ value	0.70	0.41	0.70	0.60	13
K14	H value	−0.62	−0.61	0.53	−0.24	18
K15	C value	0.70	0.40	0.71	0.61	10
K16	Ovule longitudinal diameter	0.69	0.99^a^	0.51	0.73	4
K17	Ovule transverse diameter	0.78	0.90^a^	0.59	0.76	3
K18	Ovule shape index	−0.04	−0.01	−0.47	−0.17	19
K19	Ovule fresh weight	0.84^a^	0.86^a^	0.81^a^	0.84^a^	2

a Represents statistical significance at the 0.05 level; MVCC represents the mean value of the correlation coefficients.

Integrated correlation and grey relational analyses revealed that PPH and OFW were the only physiological indices significantly positively correlated with embryo development rates in seedless grapes, with PPH demonstrating the strongest association. However, OFW measurement necessitates destructive sampling—complete ovule excision followed by gravimetric analysis on a high-precision balance. This process not only introduces operational complexity but also precludes the reuse of sampled ovules in subsequent embryo rescue trials, resulting in irreversible depletion of biological material. Given these limitations, OFW is deemed unsuitable as a nondestructive indicator for determining optimal sampling time. In contrast, PPH quantification enables noninvasive assessment while preserving ovule viability. This study therefore identifies PPH as the prioritized parameter for defining embryo rescue sampling timelines in grape embryo rescue breeding programs.

### 3.5 Construction of regression equation between PPH and seedless grape embryo development rate

**Fig. 4:**
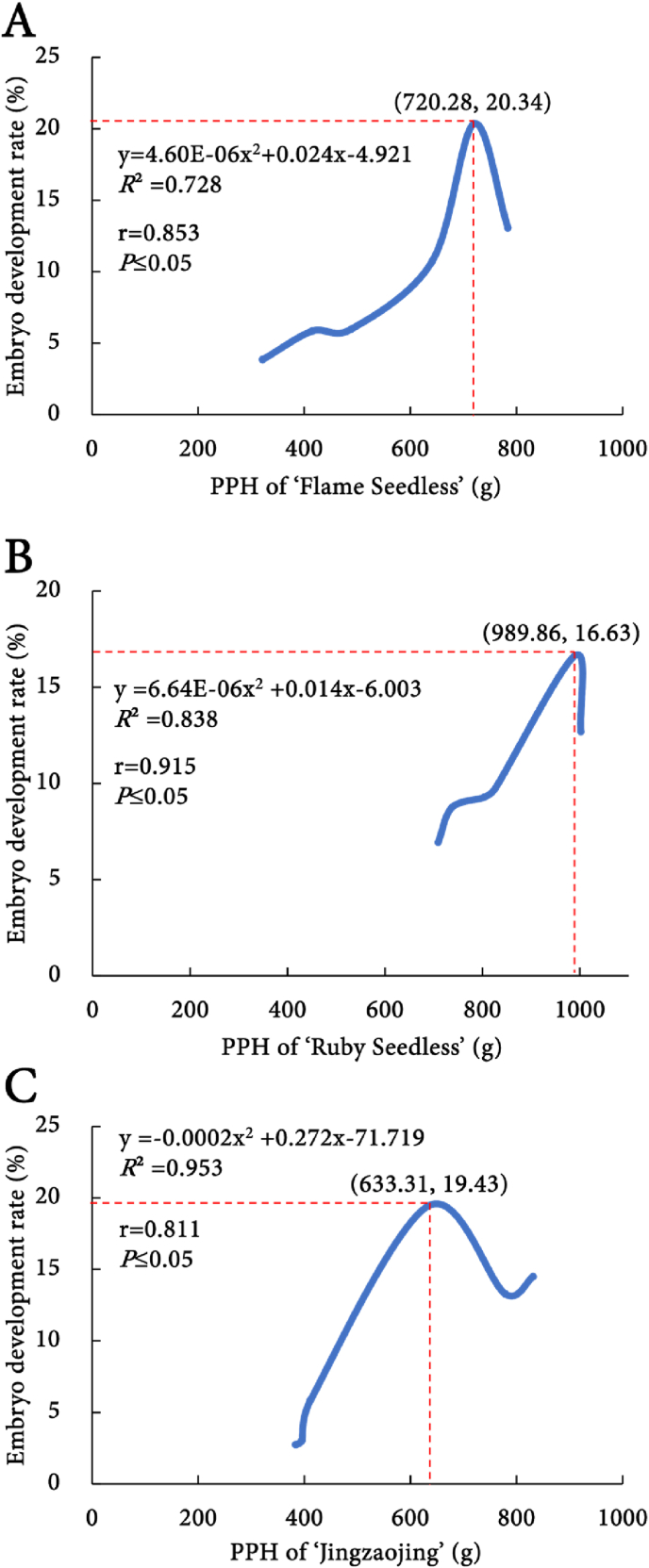
One-dimensional quadratic regression equation between the pericarp puncture hardness and embryo development rate of seedless grape.

The PPH and the embryo development rate of 'Flame Seedless' were plotted on the x-axis and the y-axis, respectively, successfully constructing the quadratic regression equation: y ​= ​4.60E-06x^2^+0.024x-4.921, *R*^2^ ​= ​0.728. Pearson correlation coefficient analysis indicated a significant correlation between the PPH of 'Flame Seedless' and its embryo development rate (r ​= ​0.853, *P* ​≤ ​0.05) (Fig. 4A). Further calculations revealed that at a PPH of 720 ​g, the embryo development rate reached its maximum, identifying this as the optimal sampling point for embryo rescue in 'Flame Seedless'.

Similarly, the PPH of 'Ruby Seedless' was plotted on the x-axis against the embryo development rate on the y-axis, yielding a quadratic regression equation: y ​= ​6.64E-06x^2^+0.014x-6.003, *R*^2^ ​= ​0.838. Pearson correlation analysis also showed a significant correlation between the PPH and embryo development rate of 'Ruby Seedless' (r ​= ​0.915, *P* ​≤ ​0.05) (Fig. 4B). Calculations indicated that at a PPH of 990 ​g, the embryo development rate peaked, establishing this as the optimal sampling point for embryo rescue in 'Ruby Seedless'.

Lastly, for 'Jingzaojing', PPH was assigned to the x-axis and its embryo development rate to the y-axis, resulting in a quadratic regression equation: y ​= ​−0.0002x^2^ +0.272x-71.719, *R*^2^ ​= ​0.953. Pearson correlation analysis revealed a significant correlation between the two variables (r ​= ​0.811, *P* ​≤ ​0.05) (Fig. 4C). Further analysis indicated that at a PPH of 633 ​g, the embryo development rate reached its peak, designating this as the optimal sampling point for embryo rescue in 'Jingzaojing'.

In summary, the embryos of 'Flame Seedless', 'Ruby Seedless', and 'Jingzaojing' were in optimal condition when their PPH measured 720 ​g, 990 ​g and 633 ​g, respectively, facilitating the acquisition of a greater number of developed embryos. Therefore, this correlation serves as a crucial reference indicator for determining the optimal sampling time for embryo rescue. To mitigate potential experimental errors, we allow a range of ±20 ​g for the PPH values associated with the optimal sampling time. In other words, when the PPH of 'Flame Seedless', 'Ruby Seedless', and 'Jingzaojing' falls within the ranges of 720 ​± ​20 ​g, 990 ​± ​20 ​g and 633 ​± ​20 ​g, respectively, these values are proposed to indicate the optimal sampling time for each cultivar. If the hardness exceeds this range, the embryos are not fully developed; conversely, if it falls below this range, the embryos have begun to abort, which is not conducive to obtaining a high embryo development rate.

### 3.6 Establishment of a non-destructive testing model for PPH

**Fig. 5:**
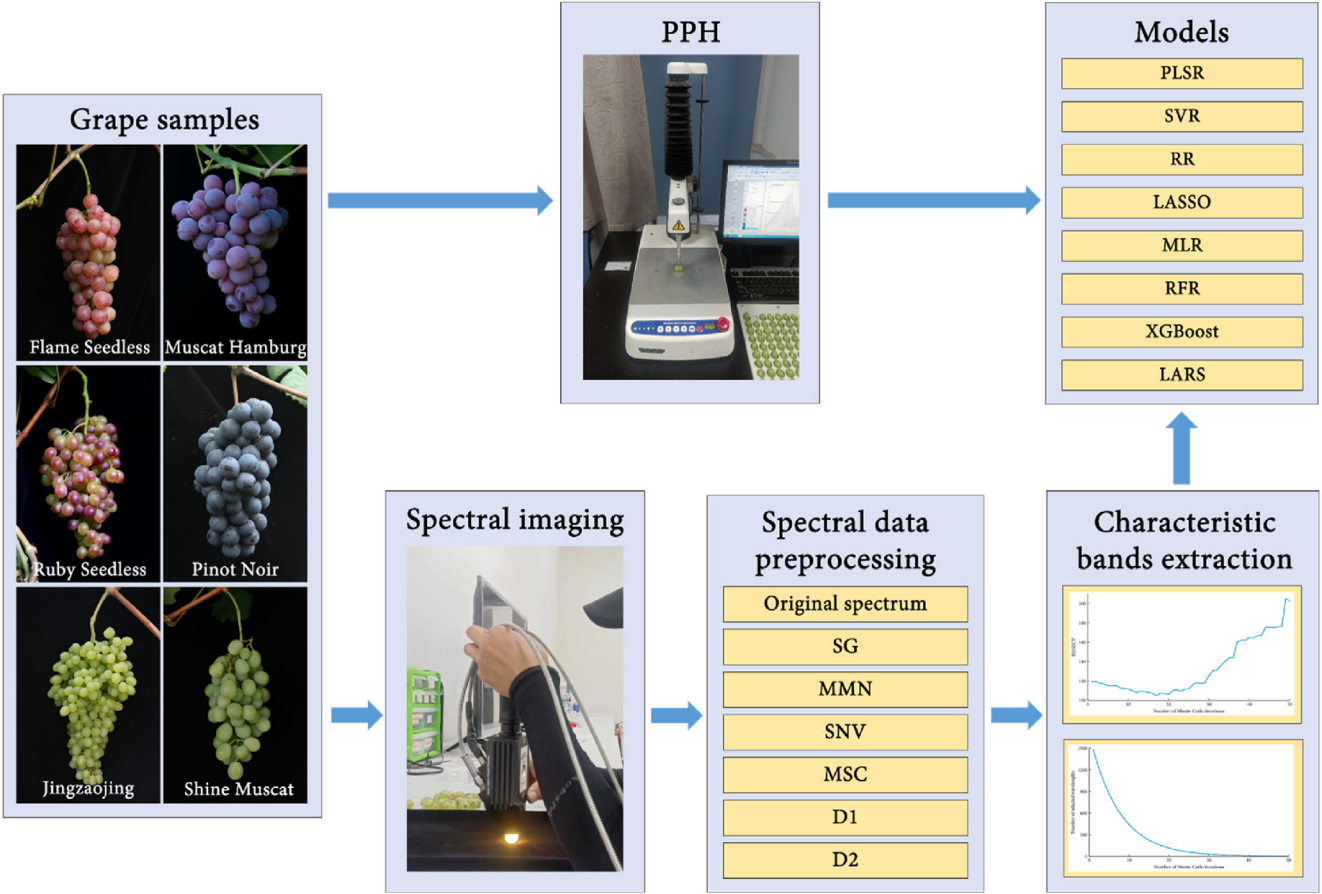
Model construction process of PPH detection based on near infrared spectroscopy combined with machine learning.

The analytical workflow commenced with preprocessing of near-infrared (NIR) spectral data, succeeded by comprehensive feature analysis and discriminative feature extraction. Eight machine learning algorithms were systematically applied to 15 stratified datasets, enabling rigorous comparative evaluation that culminated in identification of the optimal predictive model for PPH quantification (Fig. 5).

#### 3.6.1 Feature analysis and pretreatment of the near infrared spectrum of grape berry

The near-infrared spectral reflectance profiles of grapes samples with differential PPH were analyzed to characterize their optical variation patterns (Fig. S1). Due to the properties of chlorophyll, which absorbs red and blue light while reflecting green light, the spectral reflectance forms a peak around 550 ​nm in the green light range, two valleys around 440 ​nm in the blue light range and 660 ​nm in the red light range. These spectral features correspond to established vegetation reflectance patterns documented in previous studies [49,50]. Beyond 680 ​nm, reflectance intensity increased exponentially, forming a high-reflectance plateau (780–1000 ​nm) attributed to near-infrared photon scattering within the pericarp's cellular matrix. A distinct absorption trough at 970 ​nm corresponded to O–H bond stretching vibrations in water molecules, as confirmed by previous spectroscopic analyses [51]. Moisture-sensitive absorption bands at 1180 ​nm and 1400 ​nm demonstrated strong correlations with berry hydration status [52].

During the data collection process, this study promptly excluded any sample data resulting from improper handling. Post-hoc analysis using Tukey's boxplot method confirmed the normality of the dataset, with no statistical outliers detected (Fig. S2). This rigorous quality assurance ensured the integrity of the data for downstream analytical workflows.

**Fig. 6:**
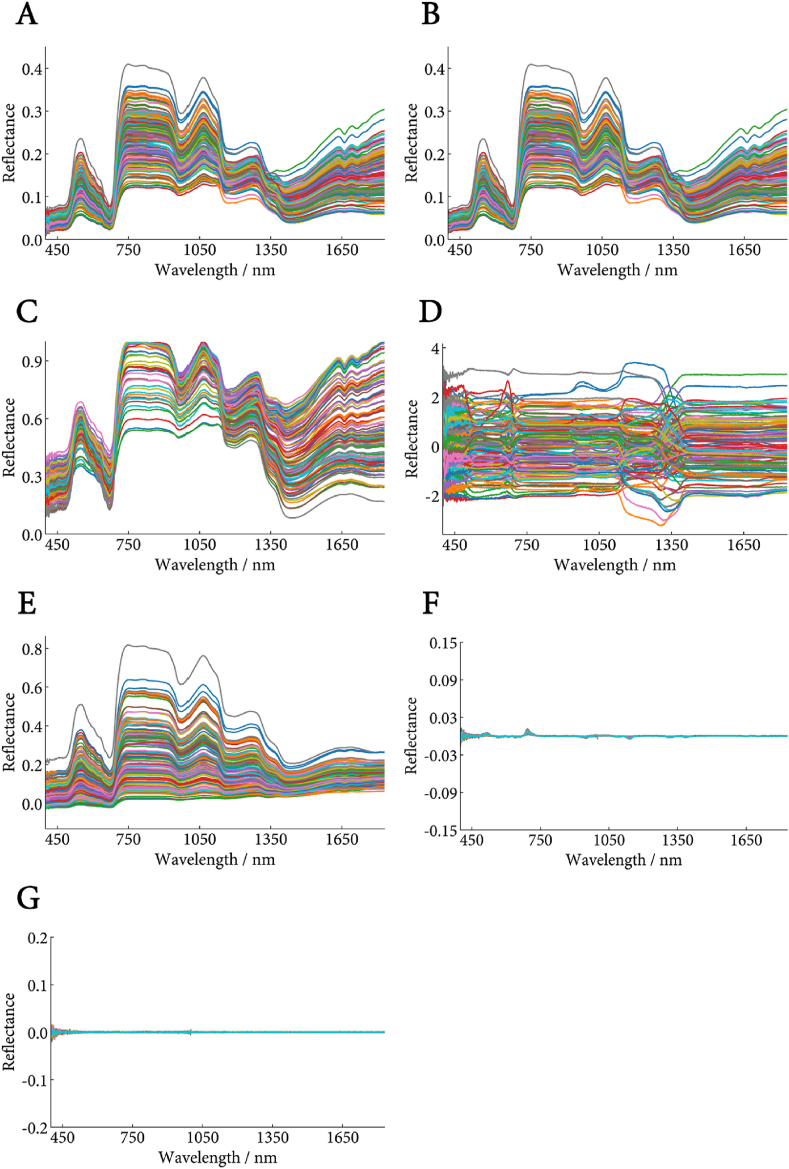
Comparison of pretreatment methods of different spectral data. (A) Original spectrum; (B) Savitzky-Golay filter; (C) Min-Max normalization; (D) Standard normal variate transformation; (E) Multiplicative scatter correction; (F) First derivative; (G) Second derivative.

Seven spectral preprocessing strategies were systematically evaluated (Fig. 6). Among the 15 data groups categorized based on the sample data, the optimal preprocessing method for 9 of these groups was D1, which includes the seedless grape cultivars 'Flame Seedless', 'Ruby Seedless', and 'Jingzaojing'. This finding suggests that the D1 method is more suitable for processing the spectral data from the grape samples in this study (Table S4). Furthermore, the Min-Max normalization method produced favorable recognition results for the 'all data' and 'seedless grape' classifications.

#### 3.6.2 Extraction of feature bands

The CARS algorithm was initialized with the full spectral range (350–1830 ​nm), configured with 50 Monte Carlo sampling iterations and 10-fold cross-validation. Through iterative optimization, the wavelength subset that achieved minimal RMSECV was identified as the optimal feature bands (Table S4). For example, when all samples are used as inputs in the CARS algorithm and the spectral preprocessing method employs the raw spectra, the rate of decrease in the number of remaining bands slows down as the sampling count increases, while the RMSECV gradually decreases (Fig. S3). When the sampling count reaches 17, the RMSECV attains its minimum value, and 171 remaining bands identified as feature bands. However, when the sampling count exceeds 30, the RMSECV sharply increases, surpassing the levels observed prior to sampling. Therefore, selecting an appropriate number of feature bands for the regression modeling of grape PPH improves modeling accuracy, while an insufficient number of selected bands may result in suboptimal modeling outcomes.

#### 3.6.3 Model construction and validation analysis of PPH for different grape cultivars

Fifteen distinct data groups were established, with each subjected to seven spectral preprocessing methods and eight modeling algorithms, generating 840 unique combinations for PPH regression modeling. Model performance was assessed using *R*^2^ and RMSE values, enabling systematic identification of optimal models per group (Table S4).

For ‘Flame Seedless’, the D1 preprocessing method demonstrated superior performance. In the training set, the models demonstrated strong performance, with the following metrics: PLSR (*R*^2^ ​= ​0.98, RMSE ​= ​22.73), RR (*R*^2^ ​= ​0.98, RMSE ​= ​22.42), MLR (*R*^2^ ​= ​0.98, RMSE ​= ​22.42), RFR (*R*^2^ ​= ​0.98, RMSE ​= ​22.22), and XGBoost (*R*^2^ ​= ​1, RMSE ​= ​0.03). In the testing set, PLSR (*R*^2^ ​= ​0.94, RMSE ​= ​42.26), RR (*R*^2^ ​= ​0.94, RMSE ​= ​43.33), and MLR (*R*^2^ ​= ​0.94, RMSE ​= ​43.33) showed commendable identification performance, with PLSR achieving the strongest generalization capacity among these models. Therefore, the D1+PLSR hybrid model was identified as optimal for 'Flame Seedless'.

**Fig. 7:**
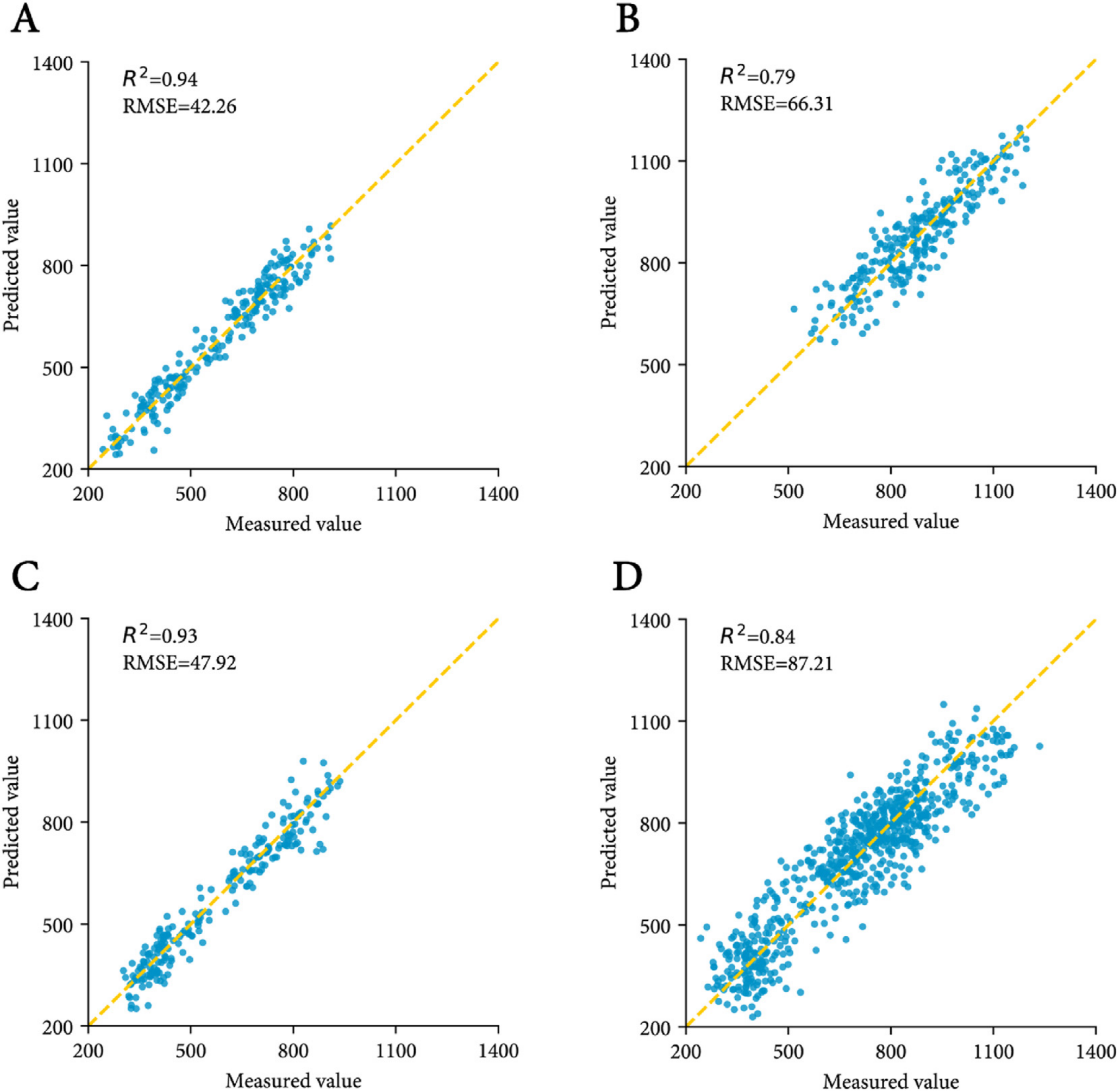
Comparison between the value predicted by the model and the true value. (A) 'Flame Seedless'; (B) 'Ruby Seedless'; (C) 'Jingzaojing'; (D) 'Seedless grape'.

For 'Ruby Seedless', the D1 preprocessing combined with RR or MLR achieved peak predictive accuracy on the test set. Given that MLR has a simpler computational model compared to RR, D1+MLR (*R*^2^ ​= ​0.79, RMSE ​= ​66.31) was selected as the optimal recognition model for 'Ruby Seedless'. 'Jingzaojing' exhibited analogous optimization patterns to 'Flame Seedless', with D1+PLSR (*R*^2^ ​= ​0.93, RMSE ​= ​47.92) showing superior performance. For all seedless grape samples, the best recognition results were obtained using MMN ​+ ​PLSR (*R*^2^ ​= ​0.85, RMSE ​= ​87.21). The optimal models for 'Flame Seedless' (D1+PLSR), 'Jingzaojing' (D1+PLSR), 'Ruby Seedless' (D1+MLR), and all seedless grape samples (MMN ​+ ​PLSR) were applied to independent test sets for PPH validation. Predictive performance was visualized through parity plots comparing measured vs. model-predicted PPH values (Fig. 7). All models showed strong agreement between predicted and measured values, with 'Flame Seedless' and 'Jingzaojing' exhibiting near-ideal fits (*R*^2^ ​> ​0.93). The optimal models for the remaining 11 data groups achieved *R*^2^ values exceeding 0.80 in the testing set, confirming methodological generalizability across diverse datasets. Notably, PLSR emerged as the preferred algorithm in 10 of these groups, demonstrating its reliability for grape PPH analysis.

Ultimately, we developed a standardized non-destructive protocol (Fig. S4) to guide optimal berry sampling for embryo rescue: 1. Parameter Pre-determination: Establish the PPH threshold range for the target cultivar during its optimal embryo rescue sampling stage (e.g., 700–740 ​g for ‘Flame Seedless’). 2. Device Pre-inspection: Ensure that the spectrometer is fully charged, complete parameter standardization and white balance calibration, and select defect-free berry samples. 3. Real-time Monitoring: Acquire NIRS data and compute real-time PPH values using cultivar-specific models. 4. Dynamic Decision-making: Immediately sample berries if the PPH values fall within the threshold range (e.g., 730 ​g). If the values exceed the threshold (e.g., 780 ​g), repeat steps 2 and 3 after 24 ​h until the optimal sampling criteria are met. This protocol ensures that embryo rescue is performed under optimal physiological conditions, significantly improving success rates.

### 3.7 Application of a non-destructive testing model for grape PPH

**Fig. 8:**
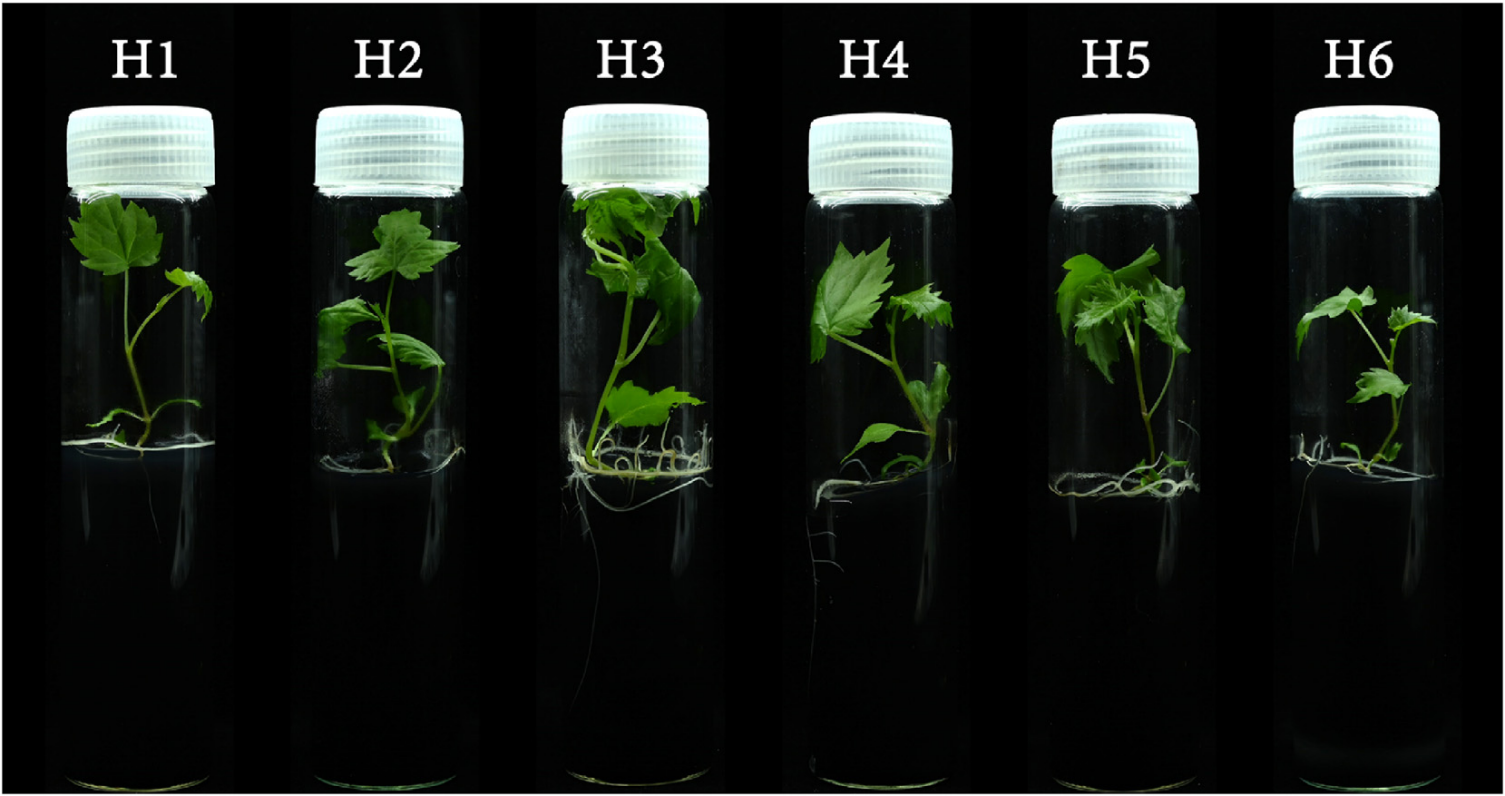
Plantlet development after embryo culture of hybrid berry 'Flame Seedless' ​× ​'Shine Muscat' collected under different PPH ranges. H1-H6 respectively represent 6 different PPH ranges of 'Flame Seedless' ​× ​'Shine Muscat' hybrid berry: H1>780 ​g, 740 ​g ​< ​H2≤780 ​g, 700 ​g ​< ​H3≤740 ​g, 660 ​g ​< ​H4≤700 ​g, 620 ​g ​< ​H5≤660 ​g, H6≤620 ​g.

Based on the optimal model for 'Flame Seedless' established in Section 3.6, NIRS was performed on berries from two hybrid crosses: 'Flame Seedless' ​× ​'Muscat Hamburg' and 'Flame Seedless' ​× ​'Shine Muscat'. This allowed for the determination of the corresponding PPH values and the implementation of embryo rescue experiments (Table 2, Fig. 8). It was found that beginning at DAP 35, PPH began to decline, while the embryo development rates and plantlet rates for both hybrid combinations gradually increased. By DAP 37, the PPH fell within the recommended sampling range for 'Flame Seedless' (700–740 ​g). Specifically, the embryo development rates for 'Flame Seedless' ​× ​'Muscat Hamburg' and 'Flame Seedless' ​× ​'Shine Muscat' were 25.19 ​% and 28.92 ​%, respectively, while the plantlet rates were 20.30 ​% and 24.90 ​%, indicating peak performance for both hybrid crosses. As time progressed, PPH continued to decrease, and the embryo development and plantlet rates began to decline rapidly (Table 2). This suggested that the optimal sampling time for embryo rescue using 'Flame Seedless' as the maternal parent was DAP 37, coinciding with the time when PPH reaches 700–740 ​g. Compared to other time points, sampling at this stage could improve the embryo development rate of 'Flame Seedless' by 7.90 ​%–21.93 ​% and the plantlet rate by 8.07 ​%–20.60 ​%.

**Table 2:** Results of embryo rescue breeding from berry collected from different crosses based on the optimal detection model of grape PPH.

Crosses	Days after pollination	PPH (g)	No. cultured ovules	No. developed embryos	Embryo development rate (%)	No. plantlets	Plantlet rate (%)
‘Flame Seedless’ ​× ​‘Muscat Hamburg’	35	>780	164	19	11.59	12	7.32
	36	740-780	229	39	17.03	28	12.23
	37	700-740	266	67	25.19	54	20.30
	38	660-700	217	34	15.67	26	11.98
	39	620-660	231	21	9.09	15	6.49
	40	≤620	205	18	8.78	11	5.37
‘Flame Seedless’ ​× ​‘Shine Muscat’	35	>780	159	24	15.09	14	8.81
	36	740-780	176	37	21.02	27	15.34
	37	700-740	249	72	28.92	62	24.90
	38	660-700	213	38	17.84	31	14.55
	39	620-660	190	22	11.58	16	8.42
	40	≤620	186	13	6.99	8	4.30
‘Ruby Seedless’ ​× ​‘Muscat Hamburg’	52	>1090	112	21	18.75	14	12.50
	53	1050-1090	126	28	22.22	21	16.67
	54	970-1010	130	41	31.54	35	26.92
	55	930-970	134	26	19.40	21	15.67
	56	890-930	123	23	18.70	17	13.82
	57	≤890	141	14	9.93	10	7.09
‘Ruby Seedless’ ​× ​‘Shine Muscat’	52	>1090	148	24	16.22	16	10.81
	53	1050-1090	170	36	21.18	26	15.29
	54	970-1010	158	47	29.75	41	25.95
	55	930-970	147	29	19.73	23	15.65
	56	890-930	149	18	12.08	13	8.72
	57	≤890	135	14	10.37	10	7.41

Based on the optimal model for 'Ruby Seedless' established in Section 3.6, NIRS was performed on the berries of the hybrid crosses: 'Ruby Seedless' ​× ​'Muscat Hamburg' and 'Ruby Seedless' ​× ​'Shine Muscat', allowing for the determination of corresponding PPH values and subsequent embryo rescue experiments. Starting at DAP 52, PPH began to decline, with the embryo development and plantlet rates for both hybrid crosses gradually improving. By DAP 54, the PPH of 'Ruby Seedless' reached the optimal sampling range (970–1010 ​g). The embryo development rates for 'Ruby Seedless' ​× ​'Muscat Hamburg' and 'Ruby Seedless' ​× ​'Shine Muscat' were 31.54 ​% and 29.75 ​%, respectively, with plantlet rates of 26.92 ​% and 25.95 ​%. Both crosses reached their peak performance, indicating that the optimal sampling time for embryo rescue with 'Ruby Seedless' as the maternal parent was DAP 54, coinciding with the point when the PPH reaches 970–1010 ​g (Table 2). Compared to other time points, sampling at this hardness level could increase the embryo development rate of 'Ruby Seedless' by 8.57 ​%–21.61 ​% and the plantlet rate by 10.26 ​%–19.83 ​%.

## 4 Discussion

The precise timing of embryo rescue sampling significantly impacts embryo development rates: sampling too early may result in underdeveloped ovules, while sampling too late can lead to embryo abortion, rendering both scenarios unsuitable for embryo culture. Conversely, sampling seedless grape ovules at their fullest developmental stage often yields higher breeding efficiency [53,54]. Currently, the optimal timing for determining the peak developmental stage of ovules is primarily assessed using DAP. This study identified DAP 37 as the ideal sampling time for the 'Flame Seedless' cultivar, while previous research has suggested optimal sampling times at various time points, including DAP 37, DAP 38, DAP 39, DAP 40, and DAP 42 [13,[20], [21], [22],^55^]. In addition, this study proposes that DAP 54 is the optimal sampling time for 'Ruby Seedless', contrasting with earlier findings that recommend DAP 55 and DAP 59 [[16], [17], [18]]. As such, variations in conclusions among different researchers regarding the optimal sampling times based on DAP are significantly influenced by factors such as the year, climatic conditions and cultivation management practices [19]. Some researchers have used the developmental status of the berry or ovule as a reference to determine the optimal timing for embryo rescue sampling [[23], [24], [25],^56^]. However, this method not only requires the destruction of the berry's structure to ascertain the optimal sampling time, leading to wastage of experimental materials, but also lacks precise evaluation criteria to define the developmental status. These shortcomings have resulted in limited recognition of this approach. To explore models for the accurate and non-destructive determination of the optimal sampling time, the present study first investigated the variations in berry and ovule characteristics among different grape cultivars, establishing a close relationship between the PPH of seedless grapes and the timing of embryo rescue sampling. Utilizing a texture analyzer capable of accurately measuring berry texture [41], we identified that the optimal sampling times for 'Flame Seedless', 'Ruby Seedless', and 'Jingzaojing' corresponded to PPH values of 720 ​± ​20 ​g, 990 ​± ​20 ​g, and 633 ​± ​20 ​g, respectively, thereby allowing for the quantification of the optimal sampling times for embryo rescue from the perspective of berry developmental status. Subsequently, we employed NIRS and machine learning techniques to build an AI model for assessing grape PPH, successfully establishing a non-destructive method for precisely determining optimal sampling times for embryo rescue in seedless grapes. This marks the first application of AI technology in seedless grape embryo rescue breeding, providing new insights into innovative methods to enhance crop breeding efficiency.

To ensure the scientific validity and rationale of this study, we conducted a comparative optimization of previous results from various perspectives. This research used 'Pinot Noir', 'Muscat Hamburg', and 'Shine Muscat' as control cultivars of seeded grapes while focusing on the seedless cultivars 'Flame Seedless', 'Ruby Seedless' and 'Jingzaojing'. Sampling was conducted strictly according to the E-L growth stage identification standards [26]. Our findings revealed that the ovules of all three seedless grape cultivars reached their maximum developmental status during the E-L-34 stage, when the grape berry began to soften and SSC started to increase. Additionally, the embryo rescue trials indicated that the highest embryo development rates were achieved at the E-L-34 stage, consistent with the findings of Grey et al. [23] and Meng et al. [24]. After investigating 19 physiological indicators related to berry development, correlation analysis and grey relational analysis consistently revealed a strong association among the PPH, ovule fresh weight and embryo development rates of the three seedless grape cultivars. This finding aligns with those of Li et al. [56], who suggested that ovule quality serves as a reference indicator for determining the optimal sampling period, thereby supporting the reliability of our results. However, we contend that PPH is the most closely related factor to the timing of embryo rescue sampling. During the development of the AI model, we compared and analyzed 840 model combinations, concluding that the D1+PLSR model performed well in most classifications. Notably, the predicted values for the seedless cultivars 'Flame Seedless' and 'Jingzaojing' closely clustered around the standard line, achieving *R*^2^ values exceeding 0.93, which indicates the excellent recognition performance of the selected optimal models. This is similar to the findings of Kanchanomai et al. [35] and Gao et al. [57], who considered PLSR the optimal model for measuring soluble solids in mature berries of 'Kyoho' and 'Red Globe' based on near-infrared spectroscopy. However, our study utilized berry developmental stages between hard and soft periods, thereby expanding the application range of the PLSR algorithm for constructing models for grapes at different developmental stages.

The primary objective of determining the precise sampling time for embryo rescue berries is to maximize embryo development and plantlet rates. For example, as demonstrated by Xu et al. [13], sampling 'Flame Seedless' ​× ​'Muscat Hamburg' at DAP 39 with PPH values of 620–660 ​g resulted in suboptimal embryogenic outcomes: 9.09 ​% embryo development rate and 6.49 ​% plantlet regeneration rate. In contrast, our optimized protocol for 'Flame Seedless' specifies DAP37 sampling at PPH 700–740 ​g, achieving significantly higher embryogenic performance: 25.19 ​% embryo development rate and 20.30 ​% plantlet rate. Although the two sampling times differ by only two days, the resulting embryo development rate and plantlet rate show significant enhancements of 16.10 ​% and 13.81 ​%, respectively. Parallel analysis of 'Ruby Seedless' ​× ​'Shine Muscat' following Chu et al. [18] showed that DAP 55 sampling (PPH 930–970 ​g) produced limited success: 19.73 ​% embryo development rate and 15.65 ​% plantlet rate. However, our research suggests sampling 'Ruby Seedless' at DAP54 (PPH 970–1010 ​g), resulting in an embryo development rate of 29.75 ​% and plantlet rate of 25.95 ​%. These reflect improvements of 10.02 ​% and 10.30 ​% in embryo development rate and plantlet rate, respectively. Therefore, we recommend sampling 'Flame Seedless' and 'Ruby Seedless' when the PPH reaches 700–740 ​g and 970–1010 ​g, respectively, to achieve higher embryo development and plantlet rates. In summary, our method of using PPH values to determine whether the berries have reached the optimal sampling time can effectively replace the traditional approach of assessing the optimal sampling time for 'Flame Seedless' and 'Ruby Seedless' based on DAP.

Certainly, this study, which employs near-infrared spectroscopy and machine learning techniques, has achieved only preliminary non-destructive and precise sampling for seedless grape embryo rescue, thereby opening new avenues for precision breeding of seedless grapes. Nonetheless, numerous problems worthy of exploration remain to be resolved in the future. For instance: (1) optimizing the model and equipment for field applications to ensure a more lightweight and streamlined operation; (2) determining whether hyperspectral imaging technology can yield superior results in the sampling process of seedless grape embryo rescue; and (3) ascertaining whether deep learning technology outperforms traditional machine learning in addressing the challenge of accurate sampling for seedless grape embryo rescue. These scientific inquiries necessitate substantial time, effort, and financial investment for further investigation.

## 5 Conclusion

For the first time, this study established a significant association between PPH and the optimal sampling time for embryo rescue in seedless grapes. Specifically, we identified that the optimal sampling times for three grape cultivars—'Flame Seedless', 'Ruby Seedless', and 'Jingzaojing'—correspond to PPH values of 720 ​± ​20 ​g, 990 ​± ​20 ​g, and 633 ​± ​20 ​g, respectively. Additionally, we developed machine learning models based on PPH specifically for embryo rescue breeding in seedless grapes, enabling non-destructive and precise sampling. This approach was shown to increase the embryo development rates by 15 ​% and plantlet rates by 14 ​%, significantly expediting the breeding process for new seedless grape cultivars.

## Data availability

The source code is distributed under the Creative Commons Attribution 4.0 international license, permitting academic use, distribution, reproduction in any medium, provided you give appropriate credit to the original authors and the source, provide a link to the Creative Commons license, and indicate if changes were made. Unless otherwise stated, the Creative Commons Public Domain Dedication (http://creativecommons.org/licenses/by/4.0) waiver applies to the data and results made available in this paper. The source code and data provided here can be accessed at https://pan.baidu.com/s/1uLY_qscSywpYQoSe8fWH_A?pwd&equals;543n.

## Author contributions:

Y.H., Y.X., and F.W. designed the study. F.W., C.L., Z.Z., Y.C., H.G., and Y.G. conducted the majority of the experiments. F.W. and C.L. assisted with the analysis of the results. G.L., T.X., Y.W., Y.X., and Y.H. provided guidance throughout the study. F.W., and C.L. wrote the manuscript, while L.B. revised the charts and the main content. All the authors approved the ultimate manuscript.

## Funding

This work was supported by Shaanxi Province Key Research and Development Plan (2023-YBNY-080), Xi'an Agricultural Technology Research and Development Project (24NYGG0031), the China Agriculture Research System of 10.13039/501100005045MOF and MARA (CARS-29-yc-3).

## Declaration of competing interest

The authors declare that they have no known competing financial interests or personal relationships that could have appeared to influence the work reported in this paper.
