# Supplementary material for: Determination of optimal sampling time of grape embryo rescue based on near infrared spectroscopy combined with machine learning
Source: Plant Phenomics. 2025 May 29;7(2):100044. doi: 10.1016/j.plaphe.2025.100044 (PMC12709896; doi:10.1016/j.plaphe.2025.100044)
Supplement: Multimedia component 1 [file mmc1.docx]

Supplementary Data

Figs. S1 to S3

Tables S1 to S4


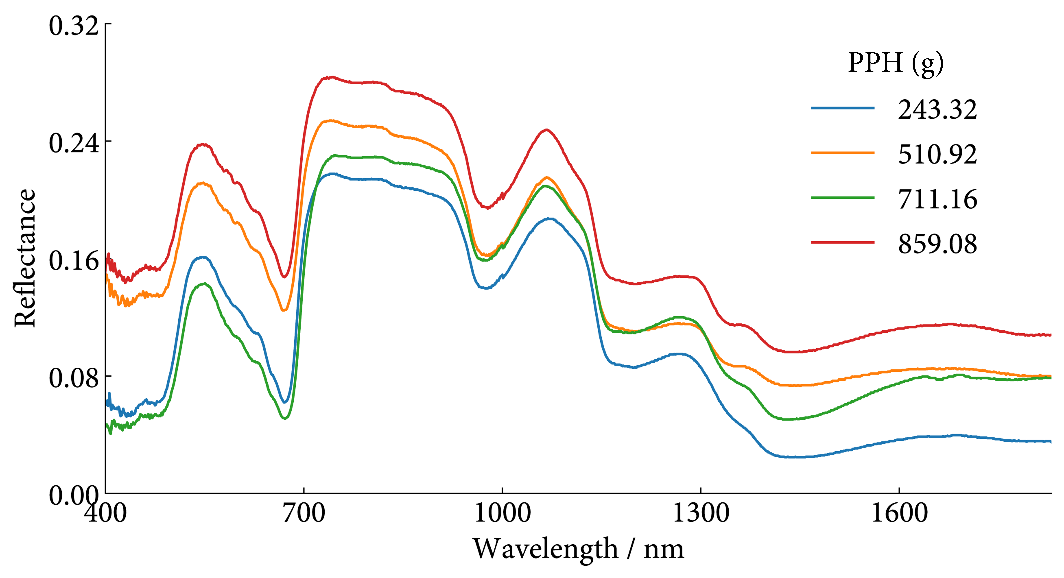


**Fig. S1.** Hyperspectral reflectance curves of grapes with different PPH.


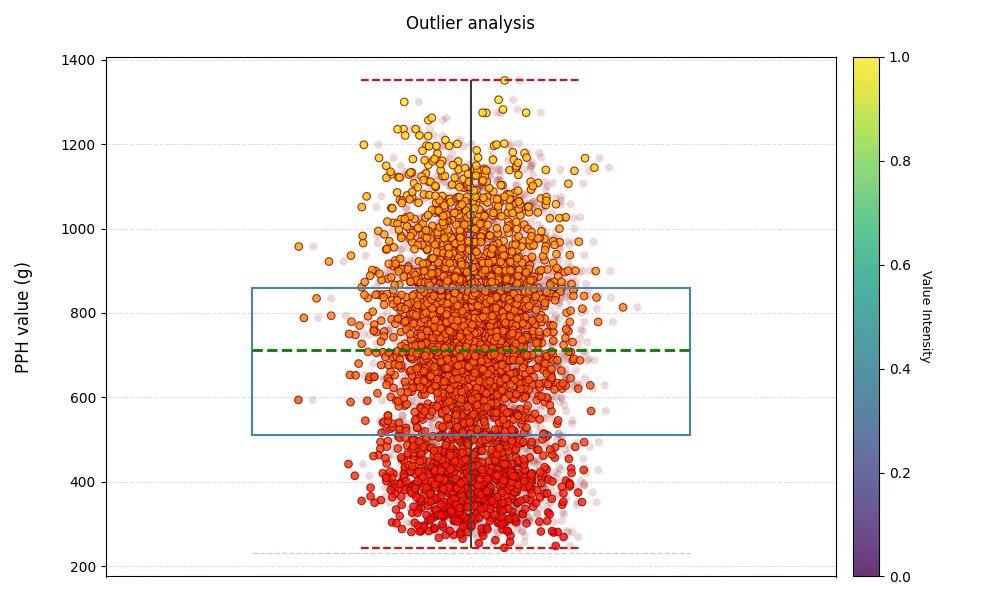


**Fig. S2.** Outlier analysis of PPH values using a boxplot


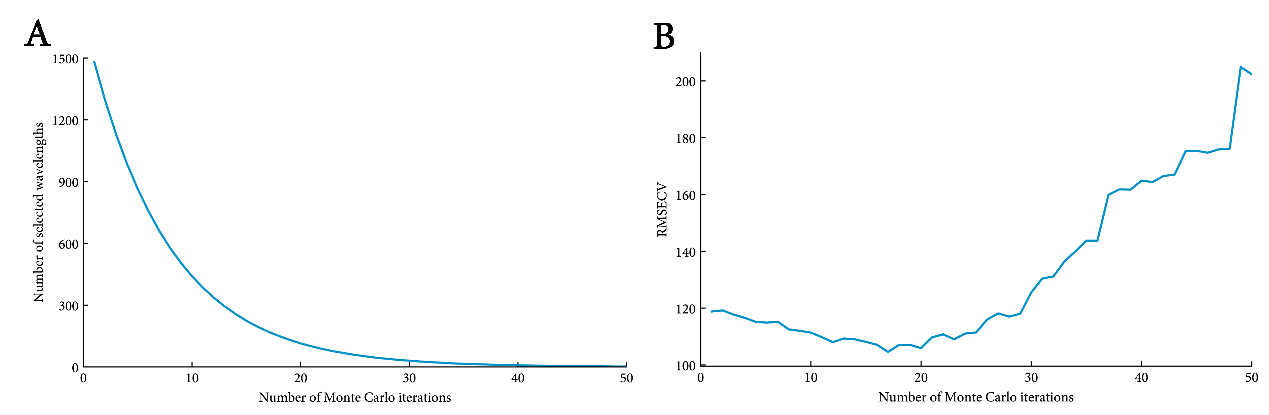


**Fig. S3.** CARS-band screening results of all samples. (A) Trends in the number of selected wavelengths; (B) RMSECV trend.


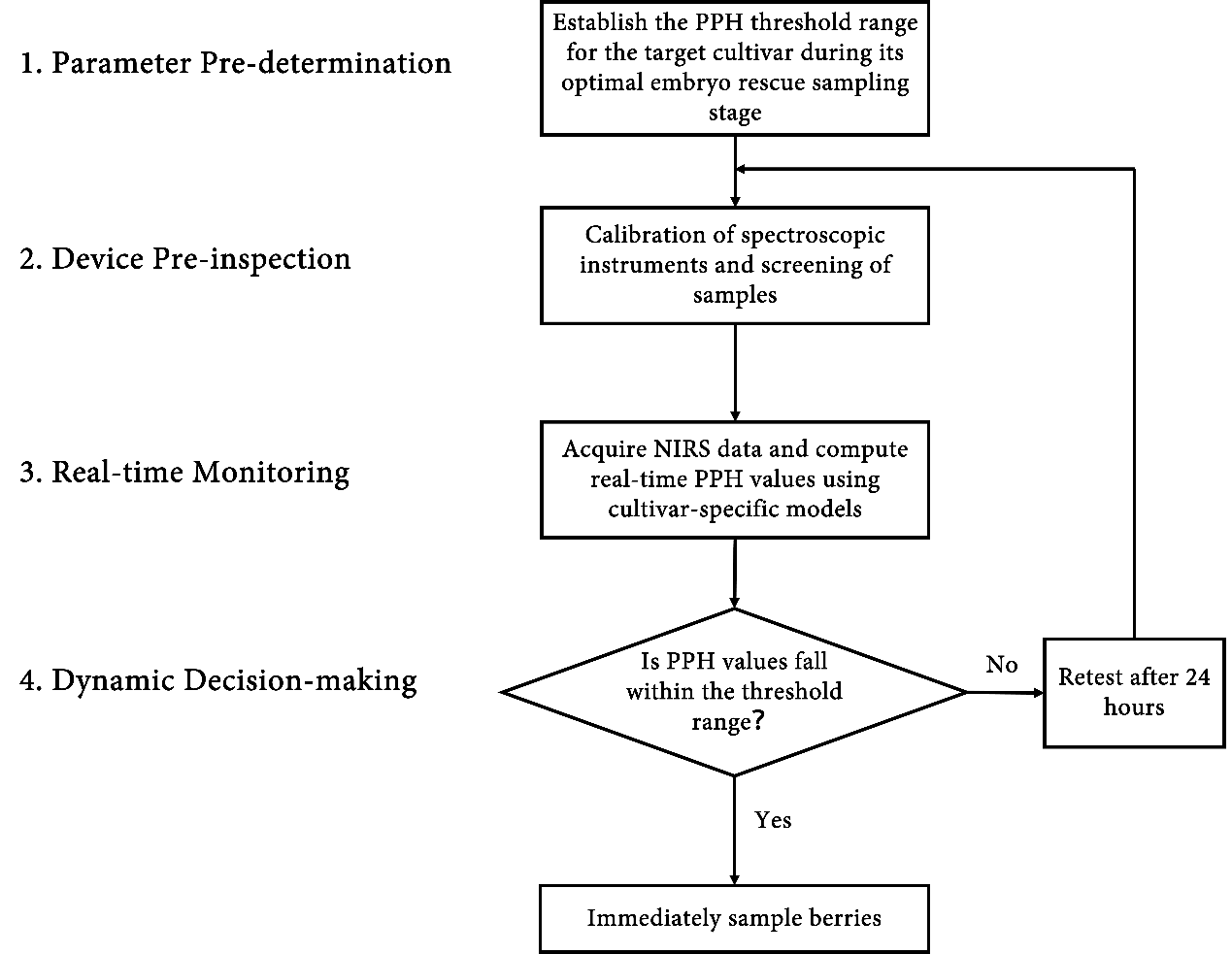


**Fig. S4.** The non-destructive and precise operational protocol based on the selected model for collecting berry samples deemed most suitable for embryo rescue experiments.

**Table S1.** Classification of NIRS data of different grape samples.

| Code | Classification | Sample | Sample numbers |
| --- | --- | --- | --- |
| 1 | Overall data | Complete sample | 2940 |
| 2 | Different cultivars | Flame Seedless | 746 |
| 3 |  | Ruby Seedless | 869 |
| 4 |  | Jingzaojing | 734 |
| 5 |  | Muscat Hamburg | 196 |
| 6 |  | Pinot Noir | 176 |
| 7 |  | Shine Muscat | 219 |
| 8 | Different batches | Batch 1 | 455 |
| 9 |  | Batch 2 | 490 |
| 10 |  | Batch 3 | 594 |
| 11 |  | Batch 4 | 481 |
| 12 |  | Batch 5 | 477 |
| 13 |  | Batch 6 | 443 |
| 14 | Different characteristics | Seedless grape | 2349 |
| 15 |  | Seeded grape | 591 |

**Table S2.** Characteristic vector and contribution rate of the principal components of each indicator.

| Code | Index | Characteristic vector | | |
| --- | --- | --- | --- | --- |
|  |  | Principal component 1 | Principal component 2 | Principal component 3 |
| K1 | Berry longitudinal diameter | 0.73 | 0.06 | 0.52 |
| K2 | Berry transverse diameter | 0.91 | 0.33 | -0.09 |
| K3 | Berry shape index | -0.42 | -0.39 | 0.74 |
| K4 | Berry fresh weight | 0.96 | 0.13 | -0.17 |
| K5 | Pericarp puncture hardness | -0.45 | 0.77 | 0.09 |
| K6 | Pericarp break distance | 0.87 | 0.13 | 0.35 |
| K7 | Pericarp stiffness | -0.84 | 0.32 | -0.17 |
| K8 | Sarcocarp firmness | -0.95 | 0.05 | -0.20 |
| K9 | Soluble solids content | 0.91 | -0.33 | -0.05 |
| K10 | CIRG value | 0.97 | 0.03 | -0.06 |
| K11 | L^*^ value | -0.98 | 0.07 | 0.05 |
| K12 | a^*^ value | 0.97 | -0.04 | -0.07 |
| K13 | b^*^ value | -0.98 | -0.09 | -0.09 |
| K14 | H value | 0.70 | -0.05 | -0.37 |
| K15 | C value | -0.98 | -0.08 | -0.10 |
| K16 | Ovule longitudinal diameter | 0.03 | 0.94 | -0.04 |
| K17 | Ovule transverse diameter | -0.02 | 0.90 | 0.30 |
| K18 | Ovule shape index | 0.18 | 0.27 | -0.81 |
| K19 | Ovule fresh weight | 0.10 | 0.96 | 0.19 |
|  | Eigen value | 11.15 | 3.82 | 2.01 |
|  | Variance contribution rate (%) | 58.66 | 20.12 | 10.58 |
|  | Cumulative contribution rate (%) | 58.66 | 78.78 | 89.36 |

**Table S3.** Grey relational analysis of the physiological and biochemical indicators of grapes at different development stages. γ represents the correlation; K1-K19 represent the specific indexes as detailed in Table S2.

| Cultivar | Stage | Correlation coefficient | | | | | | | | | | | | | | | | | | | |
| --- | --- | --- | --- | --- | --- | --- | --- | --- | --- | --- | --- | --- | --- | --- | --- | --- | --- | --- | --- | --- | --- |
|  |  | K1 | K2 | K3 | K4 | K5 | K6 | K7 | K8 | K9 | K10 | K11 | K12 | K13 | K14 | K15 | K16 | K17 | K18 | K19 |  |
| Flame Seedless | S1 | 0.78 | 0.76 | 0.99 | 0.60 | 0.95 | 0.71 | 0.66 | 0.54 | 0.70 | 0.74 | 0.94 | 0.55 | 0.68 | 0.56 | 0.68 | 0.82 | 0.87 | 0.88 | 0.84 |  |
|  | S2 | 0.70 | 0.68 | 0.81 | 0.58 | 0.93 | 0.67 | 0.79 | 0.68 | 0.62 | 0.64 | 0.92 | 0.61 | 0.80 | 0.65 | 0.80 | 0.81 | 0.84 | 0.77 | 0.92 |  |
|  | S3 | 0.51 | 0.53 | 0.50 | 0.50 | 0.60 | 0.51 | 0.57 | 0.54 | 0.49 | 0.47 | 0.56 | 0.75 | 0.58 | 0.89 | 0.59 | 0.60 | 0.59 | 0.53 | 0.72 |  |
|  | S4 | 0.70 | 0.69 | 0.75 | 0.64 | 0.81 | 0.59 | 0.96 | 0.92 | 0.71 | 0.70 | 0.78 | 0.85 | 0.92 | 0.44 | 0.93 | 0.67 | 0.69 | 0.72 | 0.66 |  |
|  | S5 | 0.66 | 0.66 | 0.73 | 0.56 | 0.88 | 0.63 | 0.94 | 0.82 | 0.63 | 0.61 | 0.82 | 0.66 | 0.93 | 0.68 | 0.90 | 0.78 | 0.81 | 0.71 | 0.89 |  |
|  | S6 | 0.58 | 0.58 | 0.65 | 0.49 | 0.86 | 0.63 | 0.93 | 0.75 | 0.49 | 0.51 | 0.75 | 0.55 | 0.99 | 0.33 | 1.00 | 0.75 | 0.75 | 0.65 | 0.97 |  |
| Ruby Seedless | S1 | 1.00 | 0.94 | 0.89 | 0.80 | 0.96 | 0.82 | 0.73 | 0.44 | 0.75 | 0.84 | 0.81 | 0.46 | 0.61 | 0.49 | 0.61 | 0.97 | 0.97 | 0.89 | 0.90 |  |
|  | S2 | 0.86 | 0.83 | 0.92 | 0.72 | 1.00 | 0.72 | 0.75 | 0.53 | 0.67 | 0.73 | 0.95 | 0.54 | 0.74 | 0.54 | 0.74 | 0.94 | 0.94 | 0.90 | 0.97 |  |
|  | S3 | 0.72 | 0.72 | 0.73 | 0.67 | 0.80 | 0.78 | 0.70 | 0.64 | 0.62 | 0.69 | 0.74 | 0.92 | 0.72 | 0.63 | 0.72 | 0.80 | 0.80 | 0.72 | 0.87 |  |
|  | S4 | 0.90 | 0.89 | 0.93 | 0.85 | 0.95 | 0.82 | 0.90 | 0.63 | 0.82 | 0.88 | 0.97 | 0.95 | 0.89 | 0.46 | 0.88 | 0.96 | 0.93 | 0.96 | 0.94 |  |
|  | S5 | 0.87 | 0.84 | 0.93 | 0.73 | 0.97 | 0.79 | 0.89 | 0.65 | 0.69 | 0.75 | 0.98 | 0.52 | 0.79 | 0.33 | 0.80 | 0.94 | 0.91 | 0.91 | 0.93 |  |
|  | S6 | 0.78 | 0.74 | 0.85 | 0.62 | 0.89 | 0.72 | 0.97 | 0.70 | 0.57 | 0.65 | 0.96 | 0.51 | 0.81 | 0.37 | 0.84 | 0.85 | 0.86 | 0.81 | 0.87 |  |
| Jingzaojing | S1 | 0.52 | 0.52 | 0.66 | 0.44 | 1.00 | 0.48 | 0.55 | 0.45 | 0.45 | 0.53 | 0.79 | 0.89 | 0.90 | 0.65 | 0.90 | 0.56 | 0.53 | 0.70 | 0.60 |  |
|  | S2 | 0.50 | 0.47 | 0.64 | 0.44 | 0.97 | 0.46 | 0.70 | 0.53 | 0.43 | 0.48 | 0.68 | 0.98 | 0.99 | 0.59 | 1.00 | 0.56 | 0.61 | 0.53 | 0.58 |  |
|  | S3 | 0.40 | 0.38 | 0.43 | 0.37 | 0.44 | 0.43 | 0.38 | 0.35 | 0.36 | 0.40 | 0.41 | 0.42 | 0.40 | 0.40 | 0.41 | 0.52 | 0.53 | 0.40 | 0.78 |  |
|  | S4 | 0.59 | 0.54 | 0.70 | 0.59 | 0.85 | 0.53 | 0.88 | 0.79 | 0.60 | 0.54 | 0.70 | 0.84 | 0.85 | 0.63 | 0.85 | 0.57 | 0.55 | 0.65 | 0.63 |  |
|  | S5 | 0.43 | 0.43 | 0.51 | 0.38 | 0.64 | 0.43 | 0.80 | 1.00 | 0.37 | 0.44 | 0.54 | 0.63 | 0.64 | 0.50 | 0.64 | 0.50 | 0.54 | 0.46 | 0.57 |  |
|  | S6 | 0.42 | 0.41 | 0.50 | 0.34 | 0.63 | 0.42 | 0.78 | 0.97 | 0.34 | 0.42 | 0.53 | 0.69 | 0.62 | 0.47 | 0.63 | 0.57 | 0.58 | 0.48 | 0.75 |  |
| γ |  | 0.66 | 0.64 | 0.73 | 0.57 | 0.84 | 0.62 | 0.77 | 0.66 | 0.57 | 0.61 | 0.77 | 0.69 | 0.77 | 0.53 | 0.77 | 0.73 | 0.74 | 0.70 | 0.80 |  |
| Ranking |  | 13 | 14 | 9 | 17 | 1 | 15 | 4 | 12 | 18 | 16 | 6 | 11 | 5 | 19 | 3 | 8 | 7 | 10 | 2 |  |

**Table S4.** Model establishment of the pericarp puncture hardness and spectral data of different grape samples. MMN, Min-Max normalization; D1, First derivative; D2, Second derivative; RMSECV, Root mean square error of cross-validation; PLSR, Partial least squares regression; SVR, Support vector regression; RR, Ridge regression; LASSO, Least absolute shrinkage and selection operator; MLR, Multiple linear regression; RFR, Random forest regression; XGBoost, eXtreme gradient boosting; LARS, Least angle regression; RMSE, Root mean square error.

| Sample | Optimal data preprocessing method | Minimum RMSECV value | Number of iterations | Optimum number of characteristic bands | Model | Modeling effect | | | |
| --- | --- | --- | --- | --- | --- | --- | --- | --- | --- |
|  |  |  |  |  |  | Training set | | Testing set | |
|  |  |  |  |  |  | *R^2^* | RMSE | *R^2^* | RMSE |
| Overall data | MMN | 96.75 | 17 | 171 | PLSR | 0.82 | 96.07 | 0.81 | 93.32 |
|  |  |  |  |  | SVR | 0.82 | 94.59 | 0.77 | 103.23 |
|  |  |  |  |  | RR | 0.83 | 94.41 | 0.8 | 96.64 |
|  |  |  |  |  | LASSO | 0.68 | 128.83 | 0.68 | 122.53 |
|  |  |  |  |  | MLR | 0.83 | 94.41 | 0.8 | 96.64 |
|  |  |  |  |  | RFR | 0.96 | 44.92 | 0.75 | 107.99 |
|  |  |  |  |  | XGBoost | 1 | 9.16 | 0.73 | 113.11 |
|  |  |  |  |  | LARS | 0.24 | 197.31 | 0.23 | 189.52 |
| Flame seedless | D1 | 41.14 | 14 | 256 | PLSR | 0.98 | 22.73 | 0.94 | 42.26 |
|  |  |  |  |  | SVR | 0.87 | 61.4 | 0.82 | 74.42 |
|  |  |  |  |  | RR | 0.98 | 22.42 | 0.94 | 43.33 |
|  |  |  |  |  | LASSO | 0.69 | 95.77 | 0.72 | 92.44 |
|  |  |  |  |  | MLR | 0.98 | 22.42 | 0.94 | 43.33 |
|  |  |  |  |  | RFR | 0.98 | 22.22 | 0.87 | 61.45 |
|  |  |  |  |  | XGBoost | 1 | 0.03 | 0.88 | 61.03 |
|  |  |  |  |  | LARS | 0.79 | 77.53 | 0.8 | 77.34 |
| Ruby seedless | D1 | 70.06 | 13 | 293 | PLSR | 0.92 | 40.25 | 0.76 | 69.57 |
|  |  |  |  |  | SVR | 0.74 | 73.86 | 0.56 | 95.61 |
|  |  |  |  |  | RR | 0.93 | 38.28 | 0.79 | 66.31 |
|  |  |  |  |  | LASSO | 0.4 | 111.41 | 0.34 | 116.03 |
|  |  |  |  |  | MLR | 0.93 | 38.28 | 0.79 | 66.31 |
|  |  |  |  |  | RFR | 0.95 | 33.05 | 0.65 | 84.44 |
|  |  |  |  |  | XGBoost | 1 | 0.13 | 0.61 | 89.71 |
|  |  |  |  |  | LARS | 0.41 | 110.7 | 0.35 | 115.21 |
| Jingzaojing | D1 | 46.33 | 13 | 293 | PLSR | 0.99 | 23.06 | 0.93 | 47.92 |
|  |  |  |  |  | SVR | 0.96 | 40.05 | 0.84 | 75.54 |
|  |  |  |  |  | RR | 0.99 | 21.93 | 0.93 | 50.67 |
|  |  |  |  |  | LASSO | 0.54 | 136.28 | 0.56 | 123.07 |
|  |  |  |  |  | MLR | 0.99 | 21.93 | 0.93 | 50.67 |
|  |  |  |  |  | RFR | 0.97 | 35.61 | 0.78 | 86.52 |
|  |  |  |  |  | XGBoost | 1 | 0.02 | 0.76 | 91.75 |
|  |  |  |  |  | LARS | 0.97 | 36.23 | 0.91 | 57.18 |
| Muscat Hamburg | D1 | 29.12 | 18 | 150 | PLSR | 1 | 5.74 | 0.98 | 36.12 |
|  |  |  |  |  | SVR | 0.97 | 45.41 | 0.78 | 119.24 |
|  |  |  |  |  | RR | 1 | 0 | 0.95 | 56.84 |
|  |  |  |  |  | LASSO | 0.81 | 105.73 | 0.76 | 126.83 |
|  |  |  |  |  | MLR | 1 | 0 | 0.95 | 56.84 |
|  |  |  |  |  | RFR | 0.98 | 34.02 | 0.81 | 113.04 |
|  |  |  |  |  | XGBoost | 1 | 0 | 0.77 | 123.95 |
|  |  |  |  |  | LARS | 0.06 | 237.4 | 0.01 | 255.28 |
| Pinot Noir | D2 | 31.78 | 18 | 149 | PLSR | 1 | 6.25 | 0.95 | 27.71 |
|  |  |  |  |  | SVR | 0.39 | 123.84 | 0.51 | 89.32 |
|  |  |  |  |  | RR | 1 | 0 | 0.84 | 50.2 |
|  |  |  |  |  | LASSO | 0.48 | 114.62 | 0.47 | 92.13 |
|  |  |  |  |  | MLR | 1 | 0 | 0.84 | 50.2 |
|  |  |  |  |  | RFR | 0.92 | 45.8 | 0.56 | 84 |
|  |  |  |  |  | XGBoost | 1 | 0 | 0.42 | 96.82 |
|  |  |  |  |  | LARS | 0.8 | 71.74 | 0.45 | 93.96 |
| Shine Muscat | D2 | 32.3 | 17 | 171 | PLSR | 1 | 8.36 | 0.95 | 46.62 |
|  |  |  |  |  | SVR | 0.8 | 85.87 | 0.67 | 117.36 |
|  |  |  |  |  | RR | 1 | 0 | 0.92 | 57.22 |
|  |  |  |  |  | LASSO | 0.63 | 117.64 | 0.62 | 125.63 |
|  |  |  |  |  | MLR | 1 | 0 | 0.92 | 57.22 |
|  |  |  |  |  | RFR | 0.95 | 43.01 | 0.71 | 110.39 |
|  |  |  |  |  | XGBoost | 1 | 0 | 0.68 | 116.26 |
|  |  |  |  |  | LARS | 0.9 | 59.6 | 0.73 | 106.5 |
| Batch 1 | D1 | 43.32 | 15 | 224 | PLSR | 0.98 | 17.68 | 0.86 | 47.14 |
|  |  |  |  |  | SVR | 0.51 | 81.45 | 0.28 | 108.29 |
|  |  |  |  |  | RR | 0.98 | 15.55 | 0.84 | 51.97 |
|  |  |  |  |  | LASSO | 0.14 | 108.69 | 0.12 | 120.33 |
|  |  |  |  |  | MLR | 0.98 | 15.55 | 0.84 | 51.97 |
|  |  |  |  |  | RFR | 0.91 | 34.79 | 0.36 | 102.24 |
|  |  |  |  |  | XGBoost | 1 | 0 | 0.3 | 107.37 |
|  |  |  |  |  | LARS | 0.13 | 109.12 | 0.11 | 120.82 |
| Batch 2 | D2 | 54.19 | 15 | 224 | PLSR | 0.97 | 23.09 | 0.84 | 51.62 |
|  |  |  |  |  | SVR | 0.91 | 38.12 | 0.61 | 80.04 |
|  |  |  |  |  | RR | 0.97 | 20.12 | 0.82 | 54.54 |
|  |  |  |  |  | LASSO | 0.37 | 99.04 | 0.38 | 101.42 |
|  |  |  |  |  | MLR | 0.97 | 20.12 | 0.82 | 54.54 |
|  |  |  |  |  | RFR | 0.93 | 34.01 | 0.51 | 89.47 |
|  |  |  |  |  | XGBoost | 1 | 0 | 0.43 | 96.52 |
|  |  |  |  |  | LARS | 0.85 | 48.06 | 0.65 | 75.95 |
| Batch 3 | D1 | 59.69 | 14 | 255 | PLSR | 0.98 | 27.01 | 0.88 | 63.18 |
|  |  |  |  |  | SVR | 0.86 | 70.94 | 0.64 | 109.61 |
|  |  |  |  |  | RR | 0.98 | 24.78 | 0.89 | 61.16 |
|  |  |  |  |  | LASSO | 0.36 | 150.54 | 0.36 | 145.03 |
|  |  |  |  |  | MLR | 0.98 | 24.78 | 0.89 | 61.16 |
|  |  |  |  |  | RFR | 0.94 | 46.02 | 0.58 | 117.04 |
|  |  |  |  |  | XGBoost | 1 | 0.02 | 0.55 | 121.82 |
|  |  |  |  |  | LARS | -0.74 | 247.44 | -1.49 | 286.73 |
| Batch 4 | D2 | 46.58 | 14 | 255 | PLSR | 0.99 | 15.12 | 0.93 | 49.21 |
|  |  |  |  |  | SVR | 0.79 | 78.19 | 0.61 | 117.48 |
|  |  |  |  |  | RR | 0.48 | 122.29 | 0.48 | 135.72 |
|  |  |  |  |  | LASSO | 0.48 | 122.16 | 0.47 | 136.81 |
|  |  |  |  |  | MLR | 0.99 | 13.4 | 0.92 | 52.76 |
|  |  |  |  |  | RFR | 0.95 | 37.93 | 0.63 | 114.25 |
|  |  |  |  |  | XGBoost | 1 | 0 | 0.64 | 112.6 |
|  |  |  |  |  | LARS | 0.77 | 80.55 | 0.68 | 106 |
| Batch 5 | D1 | 41.27 | 12 | 336 | PLSR | 0.99 | 13.87 | 0.91 | 47.22 |
|  |  |  |  |  | SVR | 0.77 | 74.76 | 0.7 | 87.77 |
|  |  |  |  |  | RR | 1 | 0 | -1.05 | 230.5 |
|  |  |  |  |  | LASSO | 0.39 | 121.23 | 0.38 | 126.47 |
|  |  |  |  |  | MLR | 1 | 0 | -1.05 | 230.5 |
|  |  |  |  |  | RFR | 0.96 | 32.7 | 0.7 | 87.37 |
|  |  |  |  |  | XGBoost | 1 | 0.01 | 0.69 | 90.11 |
|  |  |  |  |  | LARS | 0.84 | 62.1 | 0.72 | 85.78 |
| Batch 6 | D1 | 37.09 | 12 | 336 | PLSR | 1 | 11.32 | 0.92 | 42.06 |
|  |  |  |  |  | SVR | 0.85 | 64.87 | 0.66 | 88.54 |
|  |  |  |  |  | RR | 1 | 0 | 0.65 | 90.45 |
|  |  |  |  |  | LASSO | 0.53 | 114.88 | 0.48 | 109.82 |
|  |  |  |  |  | MLR | 1 | 0 | 0.65 | 90.45 |
|  |  |  |  |  | RFR | 0.96 | 33.07 | 0.74 | 77.87 |
|  |  |  |  |  | XGBoost | 1 | 0 | 0.67 | 87.11 |
|  |  |  |  |  | LARS | 0.79 | 76.87 | 0.72 | 80.15 |
| Seedless grape | MMN | 88.13 | 20 | 114 | PLSR | 0.86 | 85.2 | 0.84 | 87.21 |
|  |  |  |  |  | SVR | 0.95 | 49.92 | 0.78 | 100.66 |
|  |  |  |  |  | RR | 0.87 | 83.47 | 0.83 | 89.41 |
|  |  |  |  |  | LASSO | 0.75 | 114.95 | 0.73 | 111.86 |
|  |  |  |  |  | MLR | 0.87 | 83.47 | 0.83 | 89.41 |
|  |  |  |  |  | RFR | 0.97 | 42.41 | 0.76 | 105.5 |
|  |  |  |  |  | XGBoost | 1 | 6.24 | 0.76 | 104.55 |
|  |  |  |  |  | LARS | 0.15 | 209.91 | 0.12 | 202 |
| Seeded grape | D1 | 63.84 | 13 | 293 | PLSR | 0.99 | 23.33 | 0.91 | 63.63 |
|  |  |  |  |  | SVR | 0.94 | 51.01 | 0.7 | 115.21 |
|  |  |  |  |  | RR | 0.99 | 20.48 | 0.91 | 62.59 |
|  |  |  |  |  | LASSO | 0.56 | 138.05 | 0.57 | 138.11 |
|  |  |  |  |  | MLR | 0.99 | 20.48 | 0.91 | 62.59 |
|  |  |  |  |  | RFR | 0.96 | 41.33 | 0.76 | 102.4 |
|  |  |  |  |  | XGBoost | 1 | 0.01 | 0.73 | 110.15 |
|  |  |  |  |  | LARS | 0.72 | 109.94 | 0.68 | 119.07 |
